# Analysis of fuzzified boundary value problems for MHD Couette and Poiseuille flow

**DOI:** 10.1038/s41598-022-12110-x

**Published:** 2022-05-19

**Authors:** Imran Siddique, Muhammad Nadeem, Ilyas Khan, Raja Noshad Jamil, Mohamed A. Shamseldin, Ali Akgül

**Affiliations:** 1 Department of Mathematics, University of Management and Technology, Lahore, 54770 Pakistan; 2 Department of Mathematics, College of Science Al-Zulfi, Majmaah University, Al-Majmaah, 11952 Saudi Arabia; 3 Department of Mechanical Engineering, Faculty of Engineering and Technology, Future University in Egypt, New Cairo, 11845 Egypt; 4 Department of Mathematics, Art and Science Faculty, Siirt University, 56100 Siirt, Turkey

**Keywords:** Engineering, Mathematics and computing

## Abstract

In an uncertain atmosphere, the magnetohydrodynamics (MHD) flow in three principal flows of the third grade fluid across two parallel plates is presented. Fuzzy differential equations are constructed by manipulating dimensionless differential equations. The prime purpose of the current article is to use a semi-analytical approach fuzzy-based Adomian decomposition method to achieve numerical results for nonlinear FDEs with fuzzy boundary conditions. Triangular fuzzy numbers are used in fuzzy BCs with help of $\alpha {\text{-cut}}$ approach. This strategy is linked to the membership function. In a graphic and tabular depiction, the effect of $\alpha$ and other constraints on fuzzy velocity profiles is explored. The current findings are in good agreement with their previous numerical and analytical results in a crisp environment.

## Introduction

Scientists are paying close attention to non-Newtonian fluids because of their frequent practices in the industry, science, and, engineering such as mayonnaise, soap, cosmetics, paints, biological solutions, blood, shampoos, glues, tars, syrups, yoghurt, and other industrial materials fall into this category. As a result, researchers have given differential type^1^ fluids a higher priority. We will focus on well known third grade fluid or differential types, which have been extensively researched in a variety of flow processes^2–9^. The study of three basic streams (especially, Poiseuille, Couette, and generalized Couette flow) attracts investigators across several non-Newtonian fluids owing to their potential applications in industries and science. Injection moulding, continuous casting, die flow, plastic forming, extrusion, and asthenosphere flows are examples of unidirectional flows utilised in polymer engineering^10–13^. The study of electrically conducting liquids moving in the presence of a magnetic field is known as MHD. MHD flow across infinite parallel plates has important applications in geophysical, geothermal reservoirs, metallurgical processing, mineral industries, pumps, astrophysical, MHD generators, polymer technology, and other fields. MHD liquid is a lubricant that prevents lubricant viscosity from changing suddenly with temperature in industrial and other applications. Kamran and Siddique^14^ investigated the three flow problems (Poiseuille, Couette, and generalized Couette flow) on MHD third grade fluid across the two parallel plates with help of ADM. There is a lot of literature on this topic, such as^11,15–18^.

The ADM was first proposed by Adomian^19–21^. ADM is a procedure for solving linear and nonlinear (DEs) that is both trustworthy and efficient. The ADM has several benefits over other analytical and numerical approaches, notably the absence of perturbation, linearization, discretization, or spatial translation. ADM was utilised by Siddiqui et al.^22^ to examine the parallel plates flow of a third grade fluid and the results were compared to numerical methods. Pirzaada and Vakaaskar^23^ used fuzzy ADM to find the solution to the fuzzy heat equation. Paripour et al.^24^ evaluated the fuzzy ADM and predictor–corrector (PC) strategies for the numerical solutions of hybrid FDEs, concluding that the ADM is superior to the PC method. In addition, For squeezed flow between the two circular plates, Siddiquie et al.^25^ compared the ADM to the homotopy perturbation technique (HPM). ADM outperforms HPM, as per their observations. Biswal et al.^26^ investigated the spontaneous convection of nanofluid flow over two parallel plates by HPM in an uncertain atmosphere. TFN stands for nanoparticle volume percentage, as well as the fact that a fuzzy output is preferable to a crisp one.

In science and engineering, fluid flow is extremely important. An increase in a wide variety of issues such as magnetic effect, chemical diffusion, and heat transfer. These physical problems are then transformed into linear or nonlinear DEs after being governed. The solution of DEs is highly influenced by physical difficulties involving coefficients, geometry, initial, parameters, and boundary conditions. The coefficients, geometry, initial, parameters, and boundary conditions are not precise because of measurement errors, mechanical faults, confidence intervals, and other causes. As a result, fuzzy sets theory (FST) is a great resource for grasping the facts at hand, and it is more realistic than assuming crispness. FDEs, in particular, are useful for reducing ambiguity and clarifying physical difficulties.

Chang and Zadeh^27^ were the first to propose the concept of a fuzzy derivative (FD). On FNs, Dubois and Prade^28^ created arithmetic techniques. Trapezoidal, triangular, and Gaussian FNs are three different types of FNs. TFNs are included here for completeness' sake. In 1987 Seikkala^29^ familiarized the concept of FD. After that, Kaleva^30^ obtained FD and integration. The geometric technique for solving SFDEs was devised by Gasilov et al.^31^. To solve the second-order FDE, Khastan and Nieto^32^ used an extended differentiability approach. There were several studies a few decades ago that revolved on the topic of FDEs. Many scientists have employed FDEs to obtain well-known technological and scientific breakthroughs^33–42^.

In the literature review for third grade problems, only crisp or classic cases were considered. As a result of the above-mentioned works, In three essential flow problems of a third grade liquid across two parallel plates, we created a model to explain the fuzzy evaluation for unidirectional MHD flow. The primary purpose of this work is to use FDEs to demonstrate the uncertain flow mechanism.

The article is prepared as follows: second section contains the fundamental preliminaries; third section contains the main body of the article. In third section, the proposed study's governing equations were developed, and the governing equations were transformed to a fuzzy form for the solution by a fuzzy ADM. Fourth section presents the results and discussion in graphical and tabular formats. Fifth section contains some conclusions’.

## Preliminaries

### Definition^26^

Fuzzy set $\overline{Z}$ is defined as set of ordered pairs such that $\overline{Z} = \left\{ {\left( {x,\mu_{{\overline{Z}}} (x)} \right):x \in U,\mu_{{\overline{Z}}} (x) \in [0,\,1]} \right\},$ here $U$ is the universal set, $\mu_{{\overline{Z}}} (x)$ is membership function of $\overline{Z}$ and mapping defined as $\mu_{{\overline{Z}}} (x):U \to [0,\,1].$

### Definition^27^

$\alpha$-cut or $\alpha$-level of a fuzzy set $\overline{Z},$ defined by $Z_{\alpha } = \{ x/\mu_{{\overline{Z}}} (x) \ge \alpha \} ,$ where $Z_{\alpha }$ is crisp set and $0 \le \alpha \le 1.$

### Definition^27^

Let $\overline{Z} = (\delta ,\chi ,\eta )$ with membership function $\mu_{{\overline{Z}}} (x)$ is called a TFNs if $$\mu_{{\overline{Z}}} (x) = \left\{ {\begin{array}{*{20}l} {1 - \frac{\chi - x}{{\chi - \delta }},} \hfill & {{\text{for}}\,\,\,\delta \le x \le \chi ,} \hfill \\ {1 - \frac{x - \chi }{{\eta - \chi }},} \hfill & {{\text{for}}\,\,\,\chi \le x \le \eta ,} \hfill \\ {0,} \hfill & {{\text{otherwise}}.} \hfill \\ \end{array} } \right.$$

The TFNs with peak (center) $\chi ,$ right width $\eta - \chi > 0,$ left width $\chi - \delta > 0,$ and these TFNs are transformed into interval numbers through $\alpha$-cut approach, is written as $\overline{Z} = \left[ {v(x;\,\alpha ),\,\,u(x;\,\alpha )} \right] = \left[ {\delta + \alpha (\chi - \delta ),\,\,\eta - \alpha (\eta - \chi )} \right],$ where $0 \le \alpha \le 1.$ TFNs satisfy the following conditions: (1)$v(x;\,\alpha )$ is non-decreasing on [0,1]. (ii) $u(x;\,\alpha )$ is non-increasing on [0,1]. (iii) $u(x;\,\alpha ) \ge v(x;\,\alpha )$ on [0, 1]. (iv) $v(x;\,\alpha )$ and $u(x;\,\alpha )$ are bounded on left continuous and right continuous at [0, 1] respectively.”

### Definition^28^

Let $I^{ \mp }$ be an interval and $I^{ \mp } \subseteq R.$ A mapping $\overline{u}:I^{ \mp } \to F^{*}$ is called a fuzzy process, defined as $\overline{u}(x;\,\alpha ) = \left[ {v(x;\,\alpha ),u(x;\,\alpha )} \right],\,x \in I^{ \mp } \,$ and $0 \le \alpha \le 1.$ The derivative $\frac{{d\overline{u}(x;\,\alpha )}}{dx} \in F^{*}$ of a fuzzy process $\overline{u}(x;\,\alpha )$ is defined as $\frac{{d\overline{u}(x;\,\alpha )}}{dx} = \left[ {\frac{dv(x;\,\alpha )}{{dx}},\frac{du(x;\,\alpha )}{{dx}}} \right].$

### Definition^28^

Let $I^{ \mp } \subseteq R$ and $\overline{u}(x)$ be a fuzzy valued function define on $I^{ \mp } .$ Let $\overline{u}(x;\,\alpha ) = \left[ {v(x;\,\alpha ),u(x;\,\alpha )} \right]$ for all $\alpha$-cut. Assume that $v(x;\,\alpha )$ and $u(x;\,\alpha )$ have continuous derivatives or differentiable, for all $x \in I^{ \mp }$ and $0 \le \alpha \le 1$ then $\left[ {\frac{{d\overline{u}(x;\,\alpha )}}{dx}} \right]_{\alpha } = \left[ {\frac{dv(x;\,\alpha )}{{dx}},\frac{du(x;\,\alpha )}{{dx}}} \right]_{\alpha } .$ Similarly, we can define higher-order derivatives in the same way. Then $\left[ {\frac{{d\overline{u}(x;\,\alpha )}}{dx}} \right],$ satisfy the following conditions: (i) $\frac{du(x;\,\alpha )}{{dx}}$ and $\frac{dv(x;\,\alpha )}{{dx}}$ are continuous on [0, 1]. (ii) $\frac{dv(x;\,\alpha )}{{dx}}$ is non-decreasing on [0,1]. (iii) $\frac{du(x;\,\alpha )}{{dx}}$ is non-increasing on [0,1]. (iv) $\frac{du(x;\,\alpha )}{{dx}} \ge \frac{dv(x;\,\alpha )}{{dx}}$ on [0, 1].

## Basic equations

The following equations describe the flow of an incompressible unidirectional third-grade fluid with MHD effects:

1 $$ \nabla \cdot {\mathbf{V}} = 0. $$

2 $$ \rho \frac{{\partial {\mathbf{V}}}}{\partial t} = {\mathbf{J}} \times {\mathbf{B}} - \nabla p + div\tau^{**}, $$

where the density $$\left( \rho \right),$$ stress tensor $$\left( {\tau^{**} } \right),$$ pressure $$\left( p \right),$$ velocity vector $$\left( {\mathbf{V}} \right),$$ viscosity $$\left( \mu \right),$$ electric current $$\left( {\mathbf{J}} \right),$$ and total magnetic field $$\left( {\mathbf{B}} \right).$$ $${\mathbf{B}} = {\mathbf{B}}_{ \circ } + {\mathbf{b}}$$, where induced magnetic field $$\left( {\mathbf{b}} \right)$$ and imposed magnetic field $$\left( {{\mathbf{B}}_{o} } \right).$$ The modified Ohm's law and Maxwell's equations^14^ are applicable in the absence of displacement currents.

3 $$ {\mathbf{J}} = \sigma ^{**}\left[ {{\mathbf{E}} + {\mathbf{V}} \times {\mathbf{B}}} \right], $$

4 $$ \nabla \cdot {\mathbf{B}} = 0,\nabla \times {\mathbf{B}} = \mu_{m} {\mathbf{J}},\nabla \times {\mathbf{E}} = - \frac{{\partial {\mathbf{B}}}}{\partial t}, $$

where magnetic permeability $$\left( {\mu_{m} } \right),$$ electric field $$\left( {\mathbf{E}} \right),$$ and electrical conductivity $$\left( {\sigma^{**} } \right).$$ The MHD force in Eq. (2) can be expressed as follows:^14^

5 $$ {\mathbf{J}} \times {\mathbf{B}} = - \sigma {\mathbf{B}}_{0}^{2} {\mathbf{V}}. $$

The $$\tau^{**}$$ is given by^1–3^

6 $$ \tau ^{**} = \sum\limits_{i = 1}^{3} {\hat{S}_{i} } , $$

where $\hat{S}_{1} = \mu A_{1} ,$ $\hat{S}_{2} = \alpha_{1} A_{2} + \alpha_{2} A_{1}^{2} ,$ $\hat{S}_{3} = \beta_{1} A_{3} + \beta_{2} \left( {A_{2} A_{1} + A_{1} A_{2} } \right) + \beta_{3} A_{1} \left( {trA_{2} } \right),$ wherein, $\mu$ is coefficient of viscosity, $\alpha_{1} ,\,\,\alpha_{2} ,\,\,\beta_{1} ,\,\,\beta_{2} \,$ and $\beta_{3}$ are material constants.

Define velocity profile for one-dimensional flows as:

7 $$ {\mathbf{V}} = \left( {u\left( x \right),\,\,\,0,\,\,\,0} \right). $$

8 $$ \frac{dp^{*}}{{dx}} = 0. $$

9 $$ - \frac{dp}{{dx}} + \left( {2\alpha_{1} + \alpha_{2} } \right)\frac{d}{dx}\left( {\left( {\frac{du}{{dx}}} \right)^{2} } \right) = 0, $$

and modified pressure $p^{ \mp }$ is

10 $$ p^{ \mp } = - p + \left( {2\alpha_{1} + \alpha_{2} } \right)\left( {\left( {\frac{du}{{dx}}} \right)^{2} } \right). $$

For simplicity, the momentum Eq. (2) along with Eqs. (5)–(10) reduces to,

11 $$ - \frac{1}{\mu }\frac{{dp^{ \mp } }}{dy} + \frac{{d^{2} u}}{{dx^{2} }} + 6\frac{{\left( {\beta_{2} + \beta_{3} } \right)}}{\mu }\left( {\frac{du}{{dx}}} \right)^{2} \frac{{d^{2} u}}{{dx^{2} }} - \frac{{\sigma B_{0}^{2} u}}{\mu } = 0. $$

Equation (11) is a second-order non-linear ODE.

### The Adomian decomposition method

“Write the basic non-linear equation and discuss the basic sketch of ADM.

12 $$ L_{1} u^{ \mp } (x) + N_{1} u^{ \mp } (x) = q(x), $$

where $L_{1}$, q, and $N_{1}$ are linear, source term, and non-linear operators respectively. Also, the operator $L_{1}$ can be written as

13 $$ L_{1} = R_{1} + \hat{L}, $$

here, $\hat{L}$ is the highest order derivative in $L_{1}$ and $R_{1}$ is the remaining operator in $L_{1}$ whose order is less than the order of $\hat{L}.$ From (12) and (13) we have

14 $$ \hat{L}u^{ \mp } (x) = q(x) - R_{1} u^{ \mp } (x) - N_{1} u^{ \mp } (x). $$

Applying $$\hat{L}^{ - 1}$$

15 $$ u^{ \mp } (x) = - \hat{L}^{ - 1} R_{1} u^{ \mp } (x) - \hat{L}^{ - 1} N_{1} u^{ \mp } (x) + g(x), $$

where $g\left( {x;\alpha } \right)$ signifies the terms arising after integration of $q\left( {x;\alpha } \right)$ and calculate constants of integration with the help of boundary conditions. So, $u^{ \mp } \left( {x;\alpha } \right)$ and $N_{1} u^{ \mp } (x;\alpha )$ can be written as^19–21^,

16 $$ u^{ \mp } (x;\,\alpha ) = \sum\limits_{n = 0}^{\infty } {u_{n}^{ \mp } (x;\,\alpha )} , $$

17 $$ N_{1} u(x;\,\alpha ) = \sum\limits_{n = 0}^{\infty } {A_{n}^{*} (x;\,\alpha )} , $$

where $A_{n,\,s}^{*}$ are called Adomian polynomials^19,20^.

The algorithm of the general ADM can be communicated as

18 $$ u_{0}^{ \mp } (x) = g(x), $$

19 $$ u_{n + 1}^{ \mp } (x) = - \hat{L}^{ - 1} A_{n}^{*} (x) - \hat{L}^{ - 1} R_{1} u_{n}^{ \mp } (x),\,\,\,\,\,\,n \ge 0. $$

20 $$ u^{ \mp } (x) = \sum\limits_{k = 1}^{\infty } {u_{k}^{ \mp } (x)} . $$

In a fuzzy sense, we employ the ADM to three flow problems.

### Couette flow

**Figure 1:**
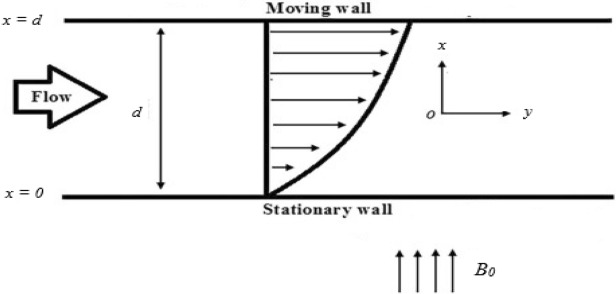
The geometry of the Couette and generalized Couette flow.

Let a third grade fluid flow steadily between two parallel plates at $x = 0$ and $x = d.$ The upper plate at $x = d$ is moving with constant velocity U while The lower plate is fixed. The magnetic field is applied vertically upward in a non-conducting manner to both plates. Also, assume that the normal flow is in y-direction while the *x*-axis is engaged as the way of flow (see in Fig. 1). When there is no pressure gradient, the consequential DE (21) with BCs (22) is^14^,

21 $$ \frac{{d^{2} u}}{{dx^{2} }} + 6\frac{{\left( {\beta_{2} + \beta_{3} } \right)}}{\mu }\left( {\frac{du}{{dx}}} \right)^{2} \frac{{d^{2} u}}{{dx^{2} }} - \frac{{\sigma B_{0}^{2} }}{\mu }u = 0, $$

22 $$ \begin{array}{*{20}l} {u\left( x \right) = 0} \hfill & {{\text{at}}\;x = 0,} \hfill \\ {u\left( x \right) = U} \hfill & {{\text{at}}\;x = d.} \hfill \\ \end{array} $$

Offering the dimensionless variables

23 $$ x^{ \mp } = \frac{x}{d},\,\,\,\beta_{2}^{ \mp } = \frac{{\beta_{2} }}{{\mu d^{2} /U^{2} }},\,\,\,u^{ \mp } = \frac{u}{U},\,\,m^{ \mp \,2} = \frac{{\sigma B_{ \circ }^{2} }}{{\mu /d^{2} }},\,\,\beta_{3}^{ \mp } = \frac{{\beta_{3} }}{{\mu d^{2} /U^{2} }}. $$

Dropping the ‘$\mp$’ the (21) and (22) becomes

24 $$ \frac{{d^{2} u}}{{dx^{2} }} + 6\beta \left( {\frac{du}{{dx}}} \right)^{2} \frac{{d^{2} u}}{{dx^{2} }} - m^{2} u = 0, $$

with the BCs

25 $$ \begin{array}{*{20}l} {u\left( x \right) = 0,} \hfill & {{\text{at}}\;x = 0,} \hfill \\ {u\left( x \right) = 1} \hfill & {{\text{at}}\;x = 1.} \hfill \\ \end{array} $$

#### For fuzzy solution

To deal with these problems, we used TFNs $(\delta ,\chi ,\eta )$ and $(d,e,f).$ Because the boundary of the parallel plates is treated as fuzzified, this discretization is applied in the boundary for certain flow behaviour. The Eqs. (24 and 25) are converted to an FDE is given below

26 $$ \frac{{d^{2} \overline{u}\left( {x;\,\alpha } \right)}}{{dx^{2} }} + 6 \odot \beta \odot \left( {\frac{{d\overline{u}\left( {x;\,\alpha } \right)}}{dx}} \right)^{2} \odot \frac{{d^{2} \overline{u}\left( {x;\,\alpha } \right)}}{{dx^{2} }} - m^{2} \odot \overline{u}\left( {x;\,\alpha } \right) = 0, $$

27 $$ \begin{array}{*{20}l} {\overline{u}(x;\,\alpha ) = \left[ {\frac{1}{100}\left( {\alpha - 1} \right),\,\frac{1}{100}\left( {1 - \alpha } \right)} \right]} \hfill & {{\text{at}}\;x = 0,} \hfill \\ {\overline{u}(x;\,\alpha ) = \left[ {\frac{1}{50}\left( {\alpha - 1} \right),\,\frac{1}{50}\left( {1 - \alpha } \right)} \right]} \hfill & {{\text{at}}\;x = 1,} \hfill \\ \end{array} $$

here lower $v(x;\,\alpha )$ and upper $u(x;\alpha )$ fuzzy velocity profiles, while (27) are fuzzy BCs^28^ are

28 $$ \frac{{d^{2} v\left( {x;\,\alpha } \right)}}{{dx^{2} }} + 6\beta \left( {\frac{{dv\left( {x;\,\alpha } \right)}}{dx}} \right)^{2} \frac{{d^{2} v\left( {x;\,\alpha } \right)}}{{dx^{2} }} - m^{2} v\left( {x;\,\alpha } \right) = 0, $$

29 $$ \begin{array}{*{20}l} {v(x;\,\alpha ) = \left( {\alpha - 1} \right)\frac{1}{100}} \hfill & {{\text{at}}\,\,x = 0,} \hfill \\ {v(x;\,\alpha ) = \left( {13\alpha - 2} \right)\frac{1}{200}} \hfill & {{\text{at}}\,\,x = 1,} \hfill \\ \end{array} $$

30 $$ \frac{{d^{2} u\left( {x;\,\alpha } \right)}}{{dx^{2} }} + 6\beta \left( {\frac{{du\left( {x;\,\alpha } \right)}}{dx}} \right)^{2} \frac{{d^{2} u\left( {x;\,\alpha } \right)}}{{dx^{2} }} - m^{2} u\left( {x;\,\alpha } \right) = 0, $$

31 $$ \begin{array}{*{20}l} {u(x;\,\alpha ) = \left( {1 - \alpha } \right)\frac{1}{100}} \hfill & {{\text{at}}\,\,x = 0,} \hfill \\ {u(x;\,\alpha ) = \left( {9 - 5\alpha } \right)\frac{1}{80}} \hfill & {{\text{at}}\,\,x = 1.} \hfill \\ \end{array} $$

The ADM is now being used in fuzzy boundary value problems Eqs. (28)–(31) and Eq. (28) becomes

32 $$ L_{1} v(x;\,\alpha ) = m^{2} v - 6\beta \left( {\frac{{dv\left( {x;\,\alpha } \right)}}{dx}} \right)^{2} \frac{{d^{2} v\left( {x;\,\alpha } \right)}}{{dx^{2} }}, $$

where $L_{1} = {{d^{2} } \mathord{\left/ {\vphantom {{d^{2} } {dx^{2} }}} \right. \kern-\nulldelimiterspace} {dx^{2} }}$ and inverse operator is $\hat{L}^{ - 1} = \iint {(.)dxdx.}$ Using $\hat{L}^{ - 1}$ in Eq. (32) we have

33 $$ v(x;\,\alpha ) = c_{1} x + c_{2} - 6\beta \hat{L}^{ - 1} \left( {\frac{dv(x;\,\alpha )}{{dx}}} \right)^{2} \frac{{d^{2} v(x;\,\alpha )}}{{dx^{2} }} + \hat{L}^{ - 1} [m^{2} v(x;\,\alpha )], $$

where the constants of integration are $c_{1}$ and $c_{2}$.

Given Eqs. (16) and (17), Eq. (33) provides

34 $$ \sum\limits_{n = 0}^{\infty } {v_{n} (x;\,\alpha )} = c_{1} x + c_{2} - 6\beta \hat{L}^{ - 1} \left( {\frac{dv(x;\,\alpha )}{{dx}}} \right)^{2} \frac{{d^{2} v(x;\,\alpha )}}{{dx^{2} }} + \hat{L}^{ - 1} [m^{2} v(x;\,\alpha )], $$

zeroth component as

35 $$ v_{0} (x;\alpha ) = c_{1} x + c_{2} , $$

and the recurrence relation as,

36 $$ v_{n + 1} (x;\,\alpha ) = \hat{L}^{ - 1} [m^{2} v_{n} (x;\,\alpha )] - 6\beta \hat{L}^{ - 1} [A_{n}^{*} (x;\,\alpha )], $$

where $$A_{n}^{*}$$ are

37 $$ \begin{aligned} A_{0}^{*} (x;\,\alpha ) & = \frac{{d^{2} v_{0} (x;\,\alpha )}}{{dx^{2} }}\left( {\frac{{dv_{0} (x;\,\alpha )}}{dx}} \right)^{2} , \\ A_{1}^{*} (x;\,\alpha ) & = \left( {\frac{{dv_{0} (x;\,\alpha )}}{dx}} \right)^{2} \frac{{d^{2} v_{1} (x;\,\alpha )}}{{dx^{2} }} + 2\frac{{dv_{0} (x;\,\alpha )}}{dx}\frac{{dv_{1} (x;\,\alpha )}}{dx}\frac{{d^{2} v_{0} (x;\,\alpha )}}{{dx^{2} }}, \\ A_{2}^{*} (x;\,\alpha ) & = \left( {\frac{{dv_{1} (x;\,\alpha )}}{dx}} \right)^{2} \frac{{d^{2} v_{0} (x;\,\alpha )}}{{dx^{2} }} + 2\frac{{d^{2} v_{0} (x;\,\alpha )}}{{dx^{2} }}\frac{{dv_{2} (x;\,\alpha )}}{dx}\frac{{dv_{0} (x;\,\alpha )}}{dx} + 2\frac{{d^{2} v_{1} (x;\,\alpha )}}{{dx^{2} }}\frac{{dv_{0} (x;\,\alpha )}}{dx}\frac{{dv_{1} (x;\,\alpha )}}{dx} \\ & \quad + \left( {\frac{{dv_{0} (x;\,\alpha )}}{dx}} \right)^{2} \frac{{d^{2} v_{2} (x;\,\alpha )}}{{dx^{2} }}, \\ & \quad \quad \quad \quad \vdots \\ \end{aligned} $$

The fuzzy BCs become

38 $$ \begin{array}{*{20}l} {v_{0} (x;\,\alpha ) = \left( {\alpha - 1} \right)\frac{1}{100},} \hfill & {{\text{at}}\,\,x = 0} \hfill \\ {v_{0} (x;\,\alpha ) = \left( {13\alpha - 2} \right)\frac{1}{200},} \hfill & {{\text{at}}\,\,x = 1,} \hfill \\ \end{array} $$

39 $$ \begin{array}{*{20}l} {v_{n} (x;\,\alpha ) = 0} \hfill & {{\text{at}}\;x = 0,} \hfill \\ {v_{n} (x;\,\alpha ) = 0} \hfill & {{\text{at}}\;x = 1,\quad n > 0.} \hfill \\ \end{array} $$

40 $$ v(x;\,\alpha ) = v_{0} (x;\,\alpha ) + v_{1} (x;\,\alpha ) + v_{2} (x;\,\alpha ) + \cdots , $$

Solving Eqs. (35) to (39) and putting all values of $v_{0} (x;\,\alpha ),\,v_{1} (x;\,\alpha ), \ldots$ in Eq. (40) we have

41 $$ v(x;\,\alpha ) = A_{1} x + A_{4} + \frac{{m^{2} }}{6}(A_{1} x^{3} + 3A_{4} x^{2} - A_{2} x)(1 - \beta A_{1}^{2} ) + \frac{{m^{4} }}{360}(3A_{1} x^{5} + 15A_{4} x^{4} - 10A_{2} x^{3} + A_{3} x) + \cdots , $$

similarly, $$u(x;\,\alpha )$$ is,

42 $$ u(x;\,\alpha ) = B_{1} x + B_{4} + \frac{{m^{2} }}{6}(B_{1} x^{3} + 3B_{4} x^{2} - B_{2} x)(1 - \beta B_{1}^{2} ) + \frac{{m^{4} }}{360}(3B_{1} x^{5} + 15B_{4} x^{4} - 10B_{2} x^{3} + B_{3} x) + \cdots . $$

### Plane Poiseuille flow

**Figure 2:**
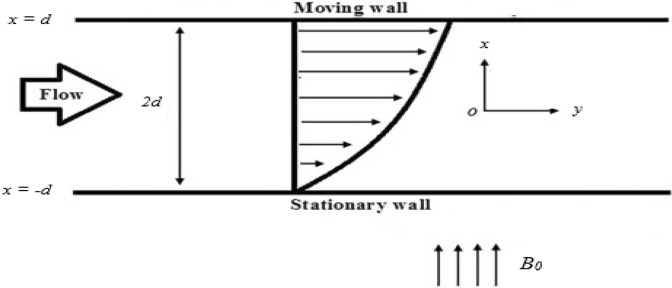
The geometry of the Poiseuille flow.

Under a constant pressure gradient, we consider the continuous laminar flow of third grade fluid among two fixed infinite parallel plates (see in Fig. 2). The gap between adjacent plates is 2*d*, are at $x = - d\,\,{\text{and}}\,\,x = d$. As a result, with the transversal magnetic field and continuous pressure gradient, the governing Eq. (11) we have

43 $$ \frac{{d^{2} u}}{{dx^{2} }} + \frac{{6\left( {\beta_{3} + \beta_{2} } \right)}}{\mu }\left( {\frac{du}{{dx}}} \right)^{2} \frac{{d^{2} u}}{{dx^{2} }} - \frac{{\sigma B_{0}^{2} }}{\mu }u = \frac{1}{\mu }\frac{{dp^{*} }}{dy}, $$

with the BCs

44 $$ \begin{array}{*{20}l} {u\left( x \right) = 0} \hfill & {{\text{at}}\,\,x = d,} \hfill \\ {u\left( x \right) = 0} \hfill & {{\text{at}}\,\,x = - d.} \hfill \\ \end{array} $$

The dimensionless parameters are presented as

45 $$ \beta _{2}^{ \mp }  = \frac{{\beta _{2} }}{{\mu d^{2} /U^{2} }},\,\,\,\beta _{3}^{ \mp }  = \frac{{\beta _{3} }}{{\mu d^{2} /U^{2} }},\,\,x^{ \mp }  = \frac{x}{d},\,\,\,m^{{ \mp 2}}  = \frac{{\sigma B_{0}^{2} }}{{\mu /d^{2} }},\,p^{ \mp }  = \frac{p}{{\mu U/d}},\,\,\,u^{ \mp }  = \frac{u}{U},\,\,y^{ \mp }  = \frac{y}{d}, $$

After dropping ‘$\mp$’ we have

46 $$ \frac{{d^{2} u}}{{dx^{2} }} + 6\beta \left( {\frac{du}{{dx}}} \right)^{2} \frac{{d^{2} u}}{{dx^{2} }} - m^{2} u = \frac{dp}{{dy}}, $$

where $\beta = \beta_{2} + \beta_{3}$, with the BCs

47 $$ \begin{array}{*{20}l} {u\left( x \right) = 0} \hfill & {{\text{at}}\,\,x = 1,} \hfill \\ {u\left( x \right) = 0} \hfill & {{\text{at}}\,\,x = - 1.} \hfill \\ \end{array} $$

Now Eqs. (46) and (47) convert in FDE as

48 $$ \frac{{d^{2} \overline{u}\left( {x;\alpha } \right)}}{{dx^{2} }} + 6 \odot \beta \odot \left( {\frac{{d\overline{u}\left( {x;\alpha } \right)}}{dx}} \right)^{2} \odot \frac{{d^{2} \overline{u}\left( {x;\alpha } \right)}}{{dx^{2} }} - m^{2} \odot \overline{u}\left( {x;\alpha } \right) = \frac{dp}{{dy}}, $$

with the fuzzy BCs

49 $$ \begin{array}{*{20}l} {\overline{u}(x;\,\alpha ) = \left[ {\frac{\alpha - 1}{{100}},\,\frac{1 - \alpha }{{100}}} \right]} \hfill & {{\text{at}}\,\,x = 1,} \hfill \\ {\overline{u}(x;\,\alpha ) = \left[ {\frac{\alpha - 1}{{50}},\,\frac{1 - \alpha }{{50}}} \right]} \hfill & {{\text{at}}\,\,x = - 1.} \hfill \\ \end{array} $$

Using the above, the solution of $v(x,\alpha )$ and $u(x,\alpha )$ are

50 $$ \begin{aligned} v\left( {x;\,\alpha } \right) & = \frac{{px^{2} }}{2} + D_{2} x + E_{2} - \frac{{\beta \left( {px + D_{2} } \right)^{4} }}{2p} - 6\beta Gx + 6\beta Fx + \frac{{2\beta^{2} }}{p}\left( {px + D_{2} } \right)^{6} + \frac{2\beta }{p}\left( {px + D_{2} } \right)^{3} \\ & \quad + m^{2} \left[ {\frac{{px^{4} }}{24} + \left( {\frac{{D_{2} - 6G\beta }}{6}} \right)x^{3} + \left( {\frac{{E_{2} + 6\beta F}}{2}} \right)x^{2} + \left( {\frac{{6Z - D_{2} }}{6}} \right)x - \frac{{p + 12E_{2} }}{24} - \frac{{\beta \left( {px + D_{2} } \right)^{6} }}{{60p^{3} }} + V} \right] \\ & \quad - m^{2} \beta \left[ \begin{gathered} \frac{{p^{3} x^{6} }}{6} + \left( {\frac{{7pD_{2}^{2} + 4pE_{2} }}{4}} \right)x^{4} + \left( {2pE_{2} D_{2} + D_{2}^{3} + 2p^{2} E_{2} + pE_{2} D_{2} } \right)x^{3} - \frac{{D_{2} \left( {px + D_{2} } \right)^{3} }}{3p} \hfill \\ + 3E_{2} D_{2}^{2} x^{2} + p^{2} D_{2} x^{5} \hfill \\ \end{gathered} \right] \\ & \quad + m^{4} \left[ {\frac{{px^{6} }}{720} + \frac{{D_{2} x^{5} }}{120} + \frac{{E_{2} x^{4} }}{24} - \frac{{D_{2} x^{3} }}{36} - \left( {\frac{{p + 12E_{2} }}{48}} \right)x^{2} } \right] - 6\beta W_{1} x - 6\beta W + \cdots \\ \end{aligned} $$

51 $$ \begin{aligned} u\left( {x;\,\alpha } \right) & = \frac{{px^{2} }}{2} + D_{3} x + E_{3} - \frac{{\beta \left( {px + D_{3} } \right)^{4} }}{2p} - 6\beta G_{1} x + 6\beta F_{1} x + \frac{{2\beta^{2} }}{p}\left( {px + D_{3} } \right)^{6} + \frac{2\beta }{p}\left( {px + D_{3} } \right)^{3} \\ & \quad + m^{2} \left[ {\frac{{px^{4} }}{24} + \left( {\frac{{D_{3} - 6G_{1} \beta }}{6}} \right)x^{3} + \left( {\frac{{E_{3} + 6\beta F_{1} }}{2}} \right)x^{2} + \left( {\frac{{6Z_{1} - D_{3} }}{6}} \right)x - \frac{{p + 12E_{3} }}{24} - \frac{{\beta \left( {px + D_{3} } \right)^{6} }}{{60p^{3} }} + V_{1} } \right] \\ & \quad - m^{2} \beta \left[ \begin{gathered} \frac{{p^{3} x^{6} }}{6} + p^{2} D_{3} x^{5} + \left( {\frac{{7pD_{3}^{2} + 4pE_{3} }}{4}} \right)x^{4} + \left( {2pE_{3} D_{3} + D_{3}^{3} + 2p^{2} E_{3} + pE_{3} D_{3} } \right)x^{3} + 3E_{3} D_{3}^{2} x^{2} \hfill \\ - \frac{{D_{3} \left( {px + D_{3} } \right)^{3} }}{3p} \hfill \\ \end{gathered} \right] \\ & \quad + m^{4} \left[ {\frac{{px^{6} }}{720} + \frac{{D_{3} x^{5} }}{120} + \frac{{E_{3} x^{4} }}{24} - \frac{{D_{3} x^{3} }}{36} - \left( {\frac{{p + 12E_{3} }}{48}} \right)x^{2} } \right] - 6\beta W_{2} x - 6\beta W_{3} + \cdots \\ \end{aligned} $$

### Generalized Couette flow

Consider a steady laminar flow of a third grade fluid among two parallel plates separated by *d* and a constant pressure gradient along the y-axis. Applied the transversal magmatic field at the upper plate in the vertically upward direction, while the lower plate is fixed (see in Fig. 1). Then consequential differential equations and boundary conditions are respected.^14^

52 $$ \frac{{d^{2} u}}{{dx^{2} }} + \frac{{6\left( {\beta_{3} + \beta_{2} } \right)}}{\mu }\left( {\frac{du}{{dx}}} \right)^{2} \frac{{d^{2} u}}{{dx^{2} }} - \frac{{\sigma B_{0}^{2} }}{\mu }u = \frac{1}{\mu }\frac{{dp^{*} }}{dy}, $$

53 $$ \begin{array}{*{20}l} {u\left( x \right) = 0} \hfill & {{\text{at}}\,\,x = 0,} \hfill \\ {u\left( x \right) = U} \hfill & {{\text{at}}\,\,x = 1.} \hfill \\ \end{array} $$

By introducing the non-dimensional parameters

54 $$ \beta_{2}^{ \mp } = \frac{{\beta_{2} }}{{\mu d^{2} /U^{2} }},\,\,\,\beta_{3}^{ \mp } = \frac{{\beta_{3} }}{{\mu d^{2} /U^{2} }},\,\,x^{ \mp } = \frac{x}{d},\,\,p^{ \mp } = \frac{{p^{*} }}{\mu U/d},\,\,\,m^{ \mp 2} = \frac{{\sigma B_{0}^{2} }}{{\mu /d^{2} }},\,\,u^{ \mp } = \frac{u}{U},\,\,y^{ \mp } = \frac{y}{d}. $$

Dropping ‘$\mp$’, we get

55 $$ \frac{{d^{2} u}}{{dx^{2} }} + 6\left( {\beta_{3} + \beta_{2} } \right)\left( {\frac{du}{{dx}}} \right)^{2} \frac{{d^{2} u}}{{dx^{2} }} - m^{2} u = \frac{dp}{{dy}}, $$

subject to the BCs

56 $$ \begin{array}{*{20}l} {u\left( x \right) = 0} \hfill & {{\text{at}}\,\,x = 0,} \hfill \\ {u\left( x \right) = 1} \hfill & {{\text{at}}\,\,x = 1.} \hfill \\ \end{array} $$

Equations (55) and (56) are now transformed to FDE in the manner of

57 $$ \frac{{d^{2} \overline{u}\left( {x;\alpha } \right)}}{{dx^{2} }} + 6 \odot \beta \odot \left( {\frac{{d\overline{u}\left( {x;\alpha } \right)}}{dx}} \right)^{2} \odot \frac{{d^{2} \overline{u}\left( {x;\alpha } \right)}}{{dx^{2} }} - m^{2} \odot \overline{u}\left( {x;\alpha } \right) = \frac{dp}{{dy}}, $$

with the fuzzy BCs

58 $$ \begin{array}{*{20}l} {\overline{u}\left( {x;\,\alpha } \right) = \left[ {\frac{\alpha - 1}{{100}},\,\frac{1 - \alpha }{{100}}} \right]} \hfill & {{\text{at}}\,\,x = 0,} \hfill \\ {\overline{u}\left( {x;\,\alpha } \right) = \left[ {\frac{\alpha - 1}{{50}},\,\frac{1 - \alpha }{{50}}} \right]} \hfill & {{\text{at}}\,\,x = 1,} \hfill \\ \end{array} $$

The Solutions of $v(x,\alpha )$ and $u(x,\alpha )$ are

59 $$ \begin{aligned} v\left( {x;\,\alpha } \right) & = \frac{{px^{2} }}{2} + \frac{\beta }{2p}\left[ {D^{4} - \left( {D^{4} - \left( {p + D} \right)^{4} } \right)x - \left( {px + D} \right)^{4} } \right] + \frac{{6\beta^{2} }}{5p}\left( {px + D} \right)^{5} + \frac{{6\beta^{2} }}{5p}\left( {px + D} \right)^{6} + Dx \\ & \quad + E\, + m^{2} \left[ \begin{gathered} - \frac{{\beta p^{2} \left( {2p + 3} \right)x^{6} }}{30} - \beta p^{2} Dx^{5} + \frac{{p - 36\beta p^{2} E - 42\beta pD^{2} }}{24}x^{4} + \frac{{D - 24\beta pED - 6D^{3} }}{6}x^{3} \hfill \\ + \frac{{\left( {2pE - 12\beta ED^{2} + \beta D^{4} } \right)x^{2} }}{4p} - \frac{p + 4D + 12E}{{24}}x + \frac{\beta }{12p}\left( {4D + 12E + p} \right)\left( {D + px} \right)^{3} \hfill \\ - \frac{{\beta \left( {D + px} \right)^{6} }}{{60p^{3} }} - \frac{\beta }{12p}\left( { - \left( {p + D} \right)^{4} + D^{4} } \right)x^{3} + Rx + \frac{{\beta D^{6} }}{{60p^{3} }} \hfill \\ \end{gathered} \right] \\ & \quad + m^{4} \left[ {\frac{{px^{6} }}{720} + \frac{{Dx^{5} }}{120} + \frac{{Ex^{4} }}{24} - \frac{p + 4D + 12E}{{144}}x^{3} } \right] + \frac{{3\beta^{2} }}{{2p^{2} }}\left( {px + D} \right)^{3} \left( {D^{4} - \left( {p + D} \right)^{4} } \right) \\ & \quad - 6\beta Ly - 6\beta K + \cdots , \\ \end{aligned} $$

similarly, a $$u(x,\alpha )$$ is,

60 $$ \begin{aligned} u\left( {x;\,\alpha } \right) & = \frac{{px^{2} }}{2} + D_{1} x + E_{1} + \frac{\beta }{2p}\left[ {D_{1}^{4} - \left( {D_{1}^{4} - \left( {p + D_{1} } \right)^{4} } \right)x - \left( {px + D_{1} } \right)^{4} } \right] + \frac{{6\beta^{2} }}{5p}\left( {px + D_{1} } \right)^{5} \\ & \quad + \frac{{6\beta^{2} }}{5p}\left( {px + D_{1} } \right)^{6} \, + m^{2} \left[ \begin{gathered} - \frac{{\beta p^{2} \left( {2p + 3} \right)x^{6} }}{30} - \beta p^{2} D_{1} x^{5} + \frac{{p - 36\beta p^{2} E_{1} - 42\beta pD_{1}^{2} }}{24}x^{4} \hfill \\ + \frac{{D_{1} - 24\beta pE_{1} D_{1} - 6D_{1}^{3} }}{6}x^{3} + \frac{{\left( {2pE_{1} - 12\beta E_{1} D_{1}^{2} + \beta D_{1}^{4} } \right)x^{2} }}{4p} \hfill \\ - \frac{{p + 4D_{1} + 12E_{1} }}{24}x + \frac{\beta }{12p}\left( {p + 4D_{1} + 12E_{1} } \right)\left( {px + D_{1} } \right)^{3} \hfill \\ - \frac{{\beta \left( {px + D_{1} } \right)^{6} }}{{60p^{3} }} - \frac{\beta }{12p}\left( {D_{1}^{4} - \left( {p + D_{1} } \right)^{4} } \right)x^{3} + R_{1} x + \frac{{\beta D_{1}^{6} }}{{60p^{3} }} \hfill \\ \end{gathered} \right] \\ & \quad + m^{4} \left[ {\frac{{px^{6} }}{720} + \frac{{D_{1} x^{5} }}{120} + \frac{{E_{1} x^{4} }}{24} - \frac{{p + 4D_{1} + 12E_{1} }}{144}x^{3} } \right] + \frac{{3\beta^{2} }}{{2p^{2} }}\left( {px + D_{1} } \right)^{3} \left( {D_{1}^{4} - \left( {p + D_{1} } \right)^{4} } \right) \\ & \quad - 6\beta L_{1} x - 6\beta K_{1} + \cdots \\ \end{aligned} $$

## Results and discussion

In a fuzzy environment, discuss the three elementary flow problems of a third grade fluid such as plane Poiseuille, Couette, and generalised Couette flow. The governing equations convert into FDEs for the analytical solutions using fuzzy ADM to find the fuzzy velocity profiles of a third-grade differential type fluid among two parallel plates with MHD (magnetic parameter *m*) effect under a constant pressure gradient $\left( {dp/dy = p} \right).$ Figures 3, 4, 5, 6, 7, 8, 9, 10, 11, 12, 13, 14, 15, 16, 17, 18, 19, 20, 21, 22, 23, 24, 25, 26, 27, 28, 29, 30, 31, 32, 33, 34, 35, 36, 37, 38, 39, 40, 41, 42 and 43 display the obtained fuzzy flow rate for various $\alpha$-cut levels $\left( {\alpha = 0,\,\,0.3,\,\,0.7,\,\,1} \right).$

**Figure 3:**
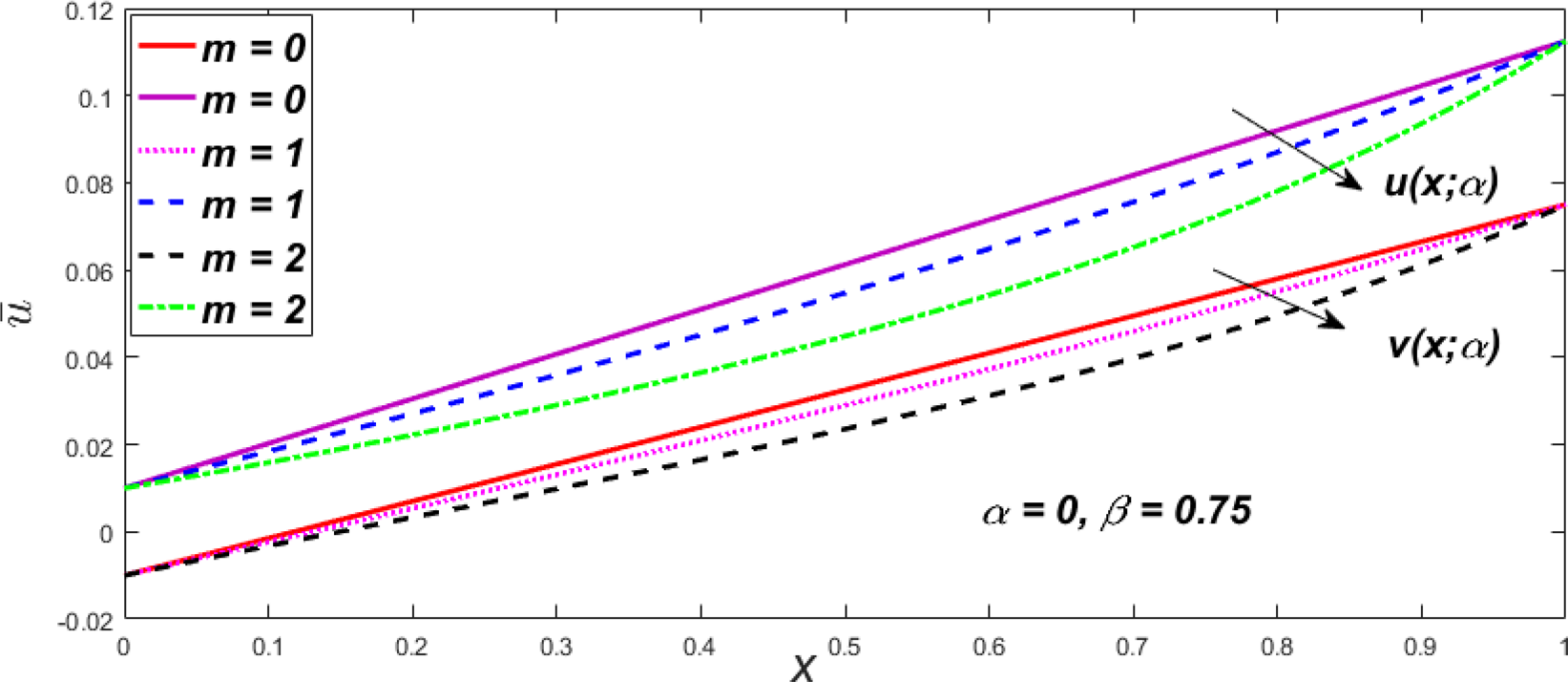
Fuzzy velocity profiles for the impact of *m.*

**Figure 4:**
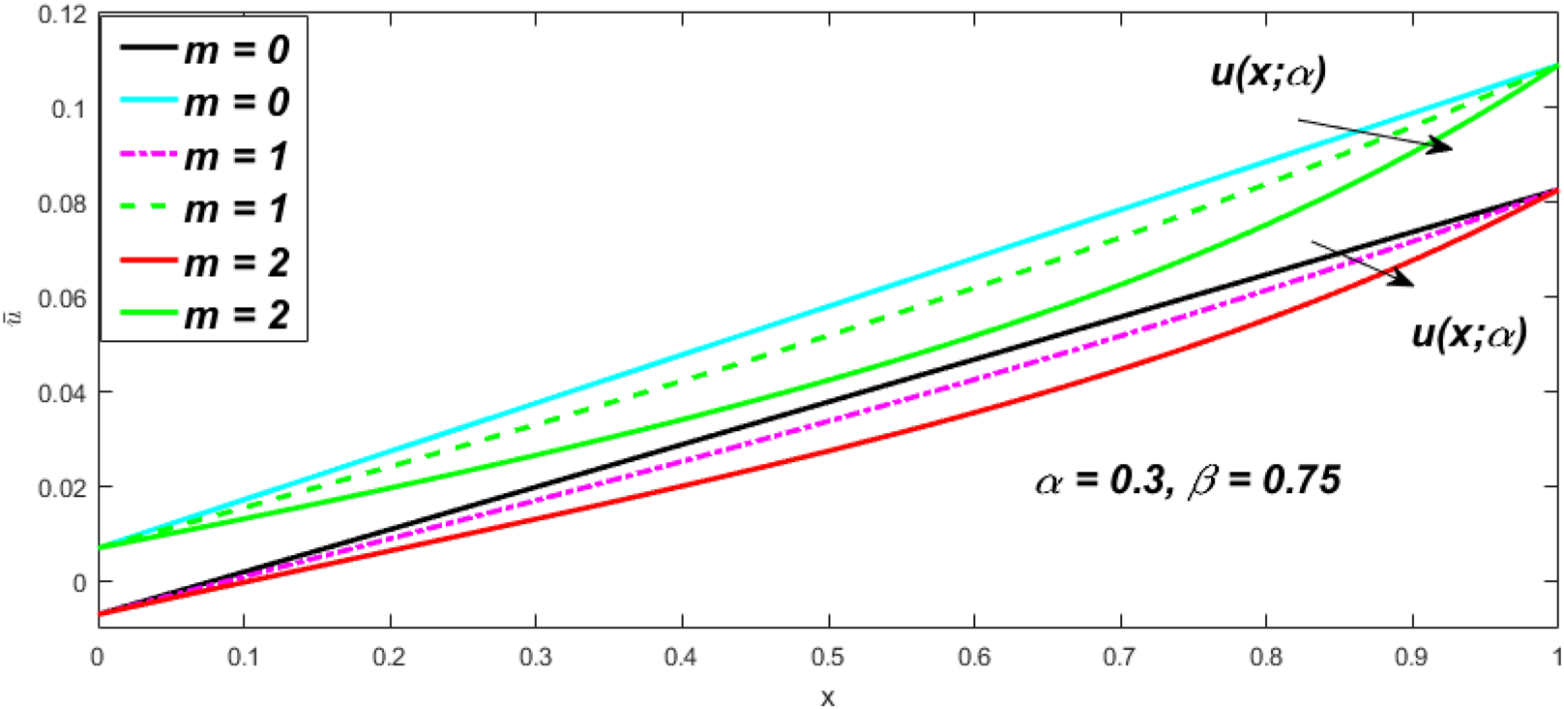
Fuzzy velocity profiles for the impact of *m.*

**Figure 5:**
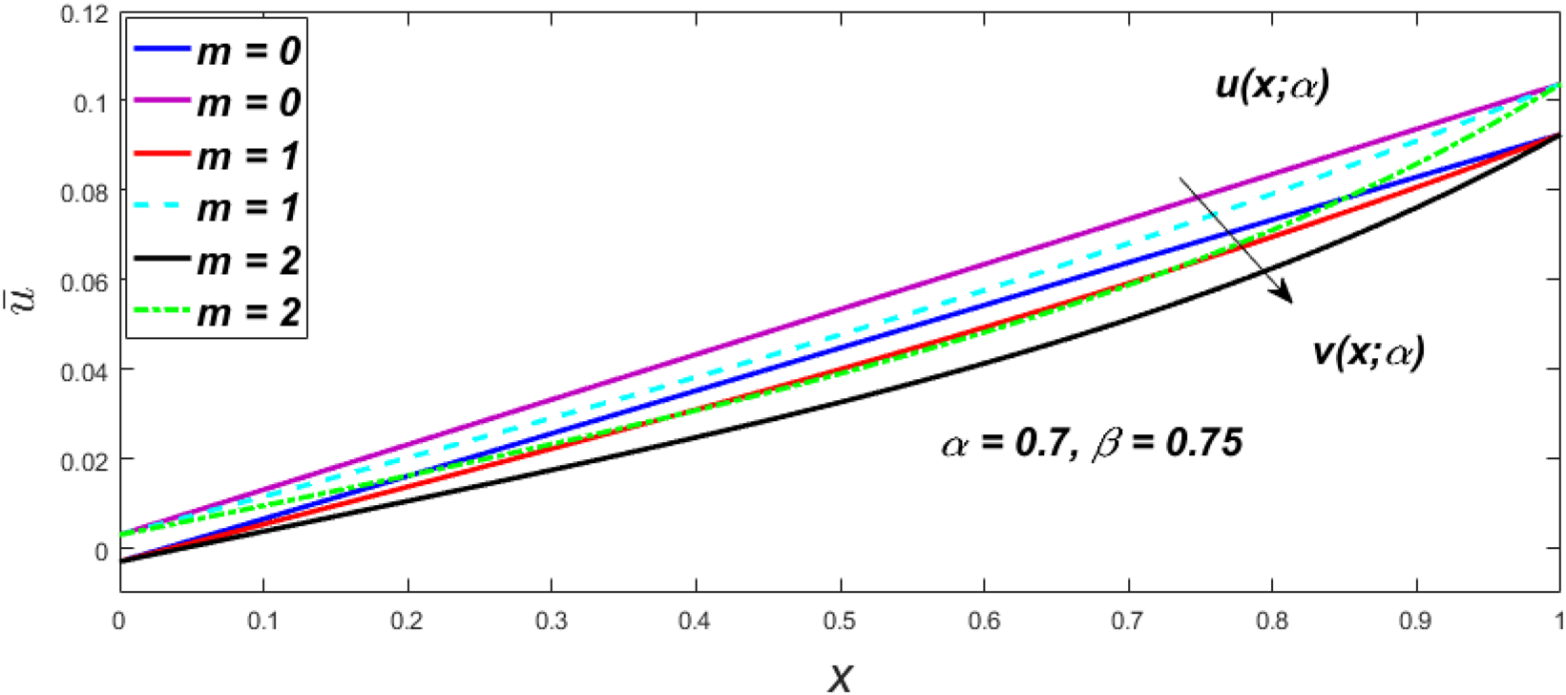
Fuzzy velocity profiles for the influence of *m.*

**Figure 6:**
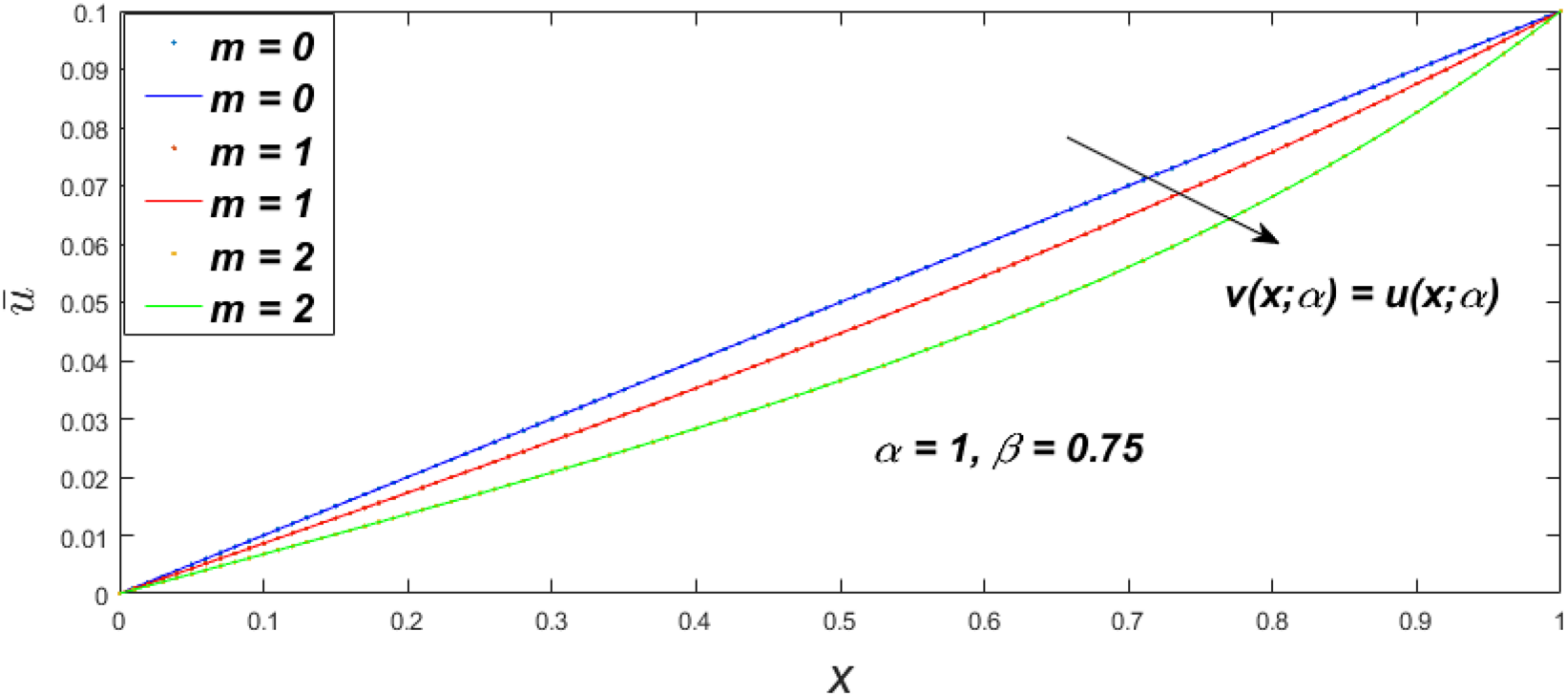
Fuzzy velocity profiles for *the* impact of *m.*

**Figure 7:**
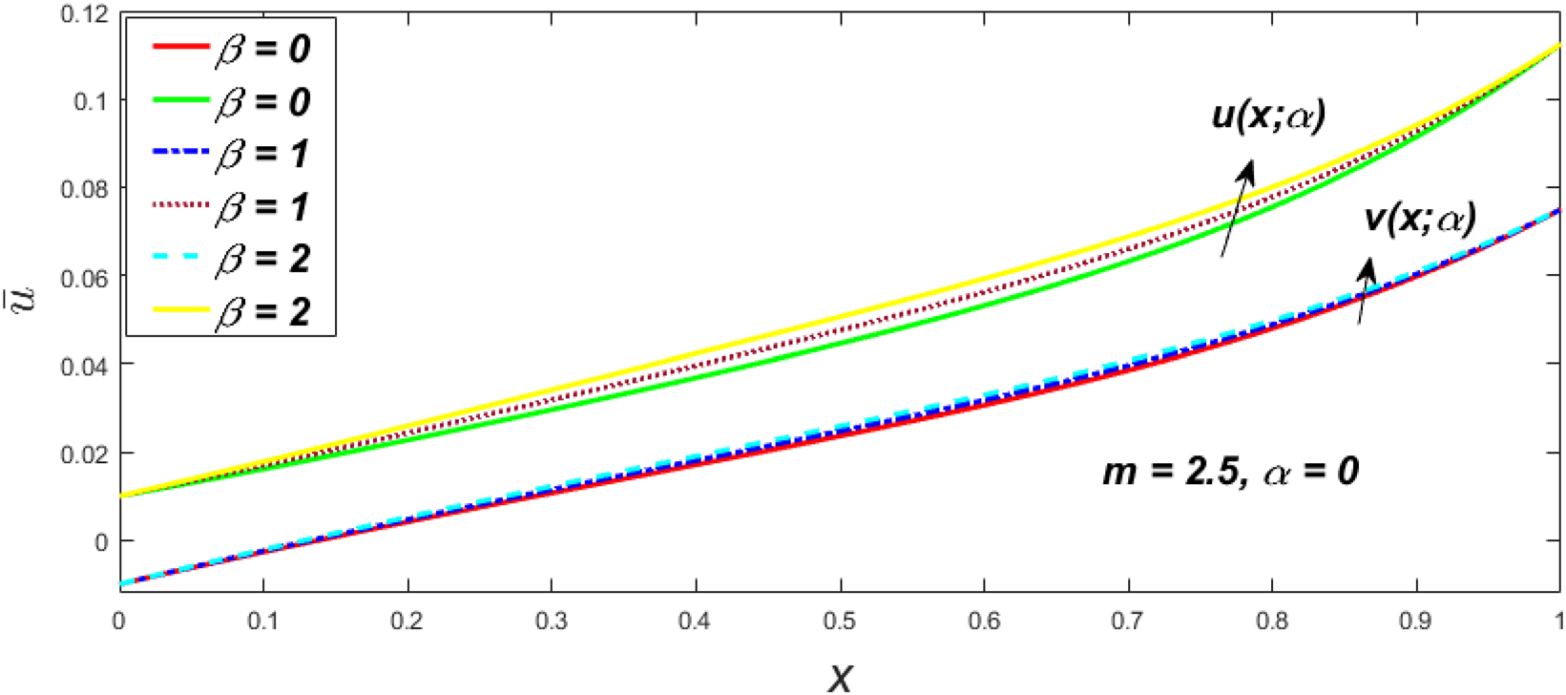
Fuzzy velocity profiles for the impact of $\beta .$

**Figure 8:**
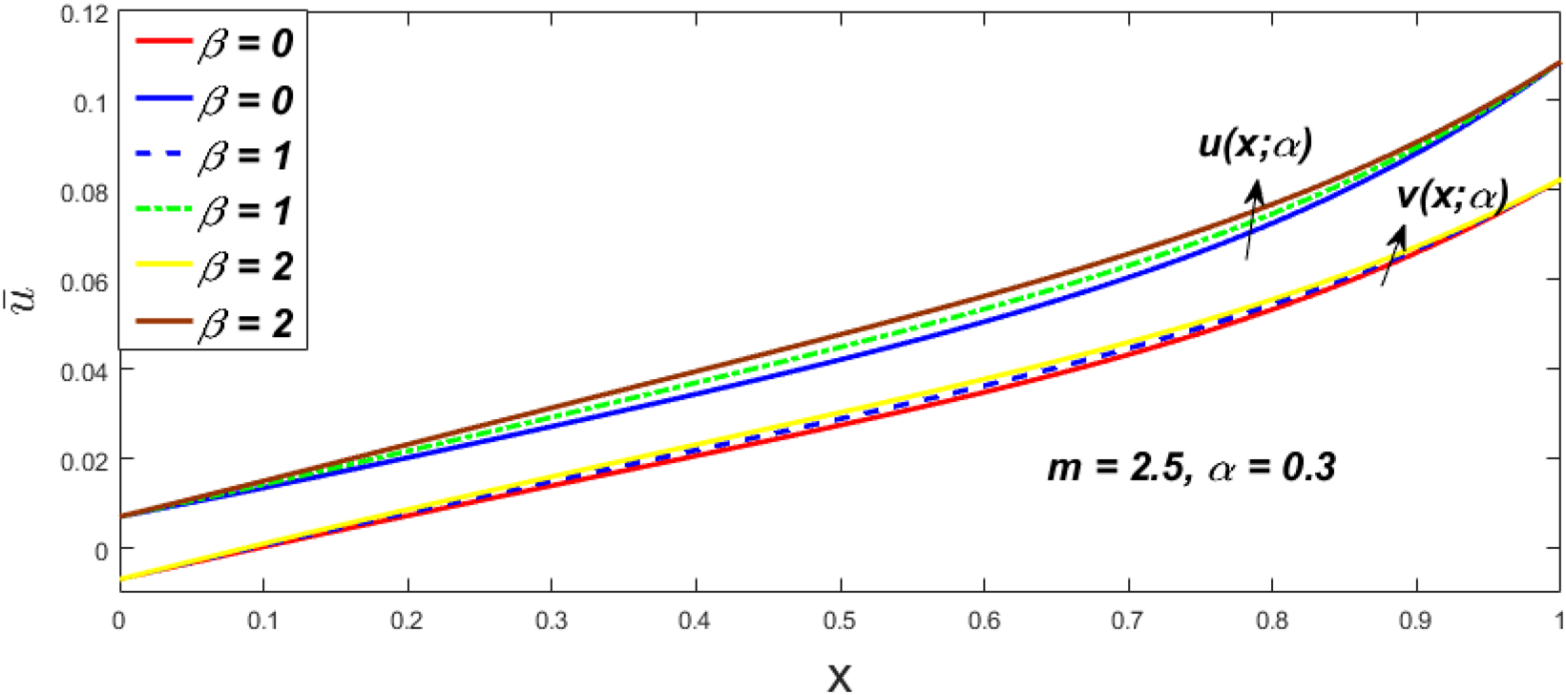
Fuzzy velocity profiles for the impact of $\beta .$

**Figure 9:**
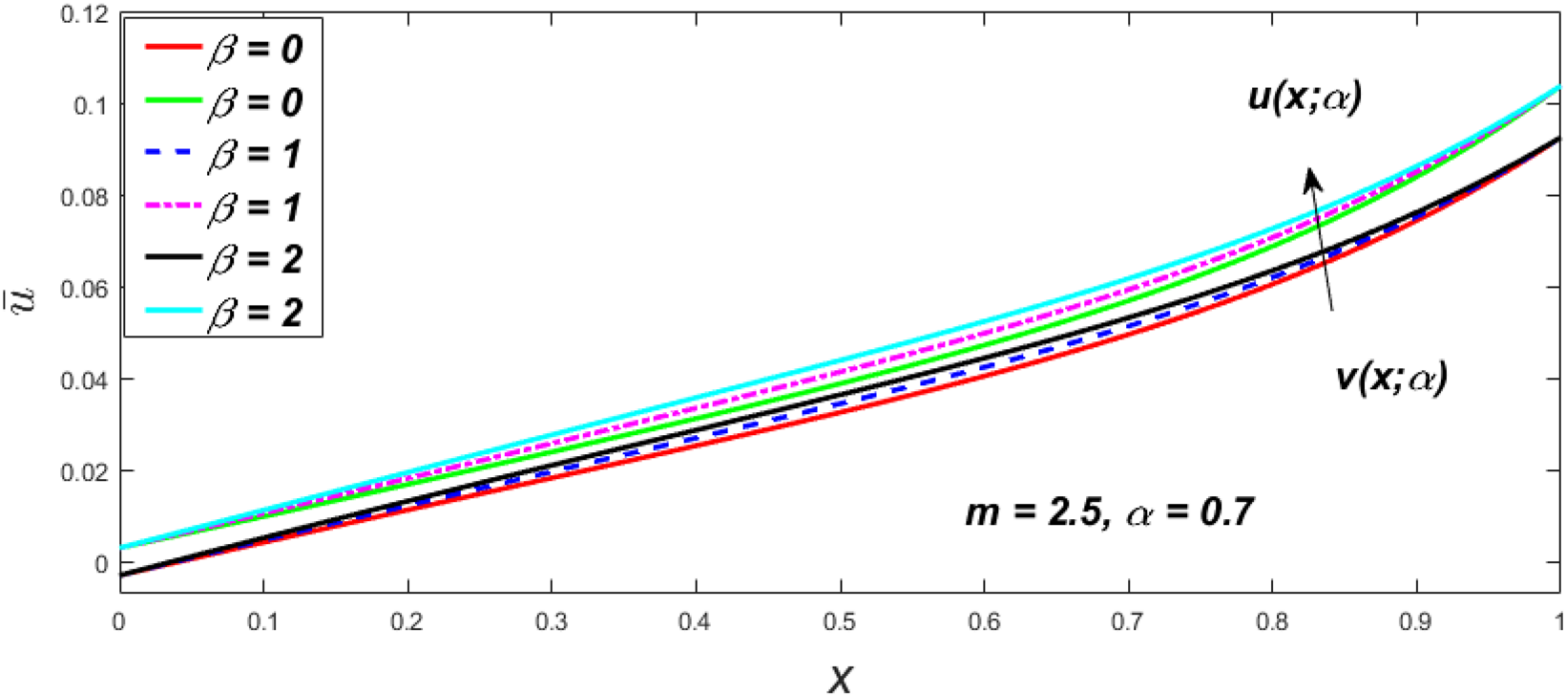
Fuzzy velocity profiles for the impact of $\beta .$

**Figure 10:**
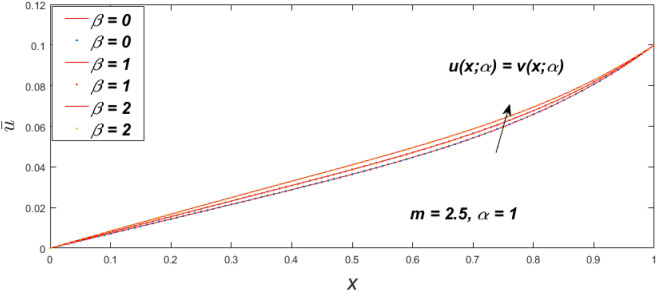
Fuzzy velocity profiles for the impact of $\beta .$

**Figure 11:**
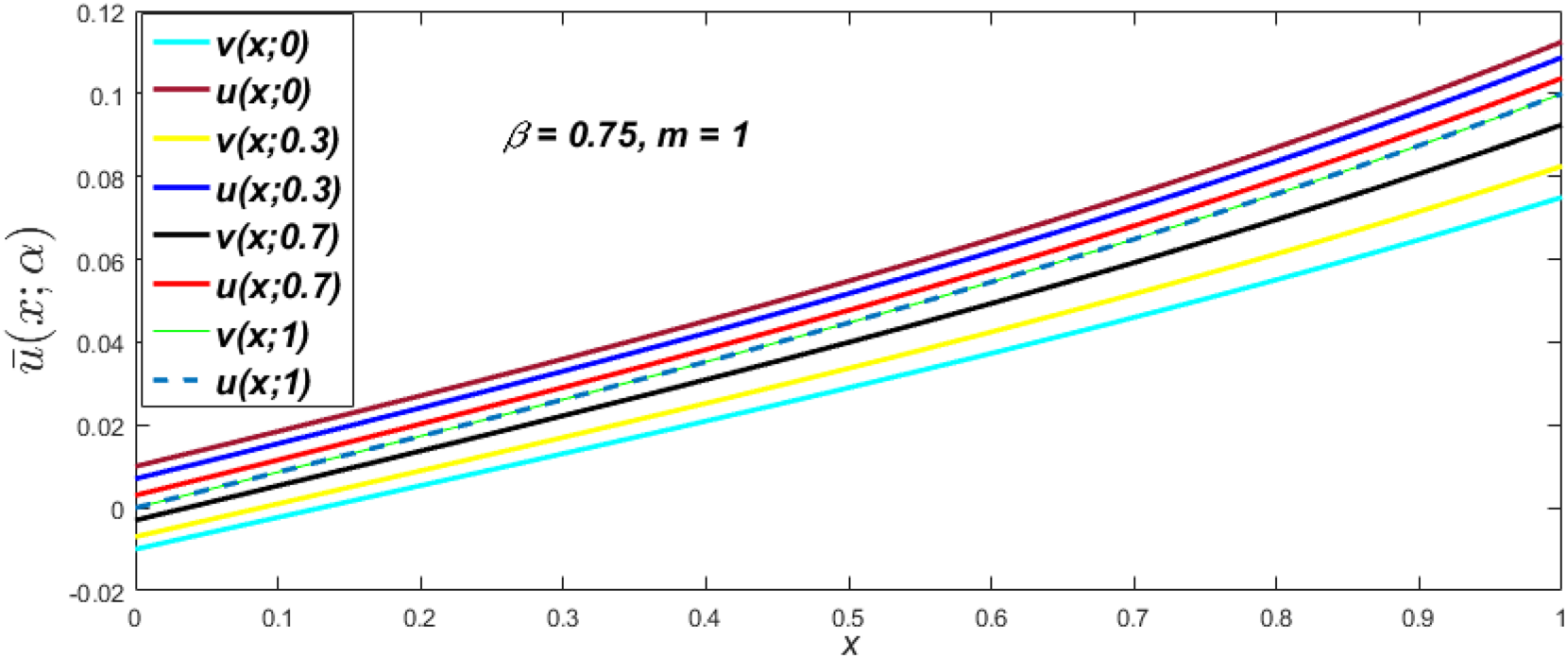
Fuzzy velocity profiles for different values of $\alpha$-cut.

**Figure 12:**
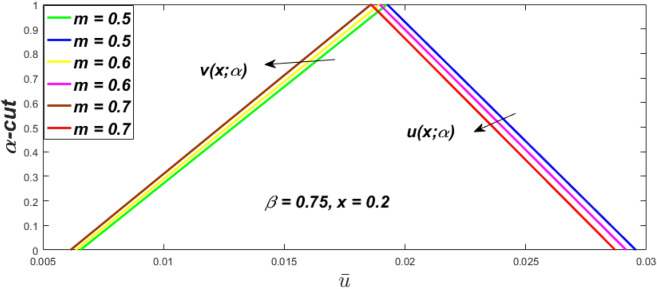
Triangular MF of fuzzy velocity profiles for the impact of *m*.

**Figure 13:**
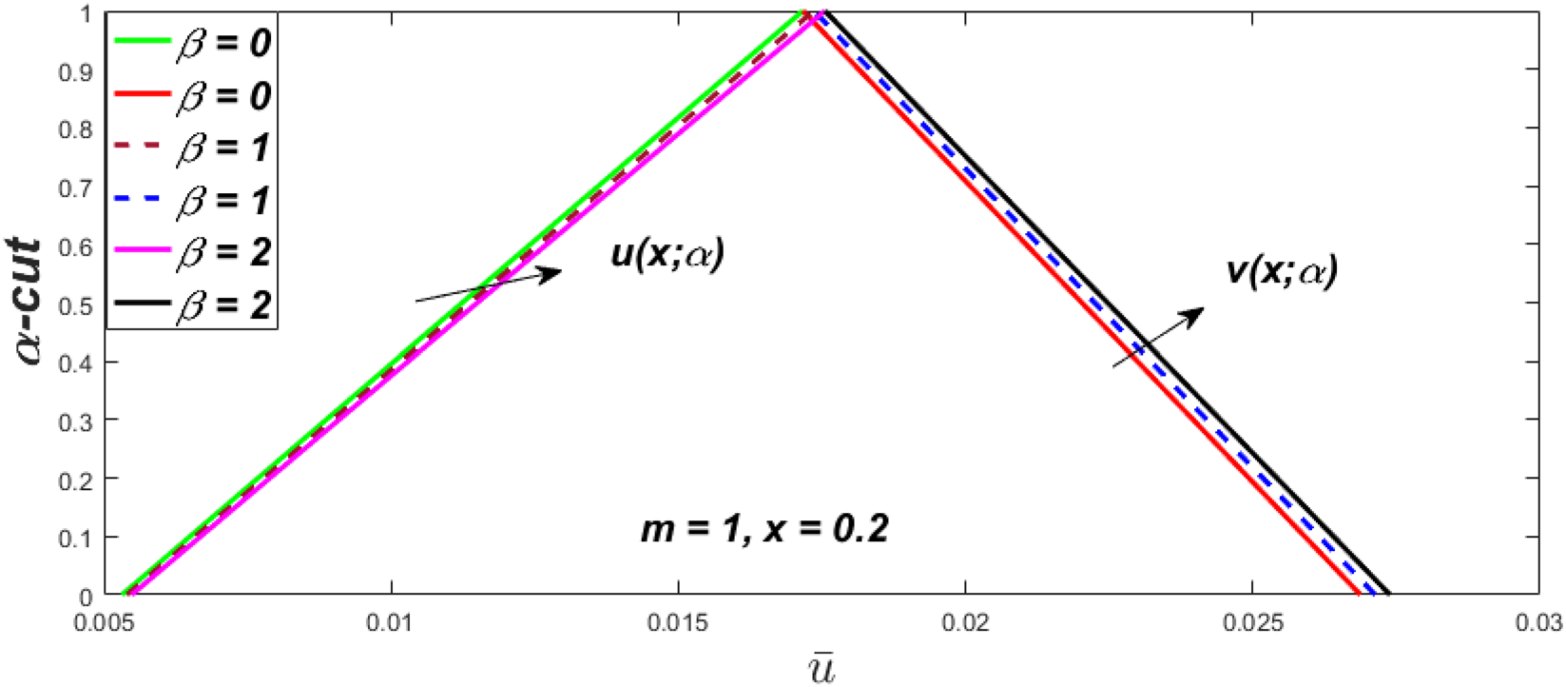
Triangular MF of fuzzy velocity profiles for impact of $\beta .$

**Figure 14:**
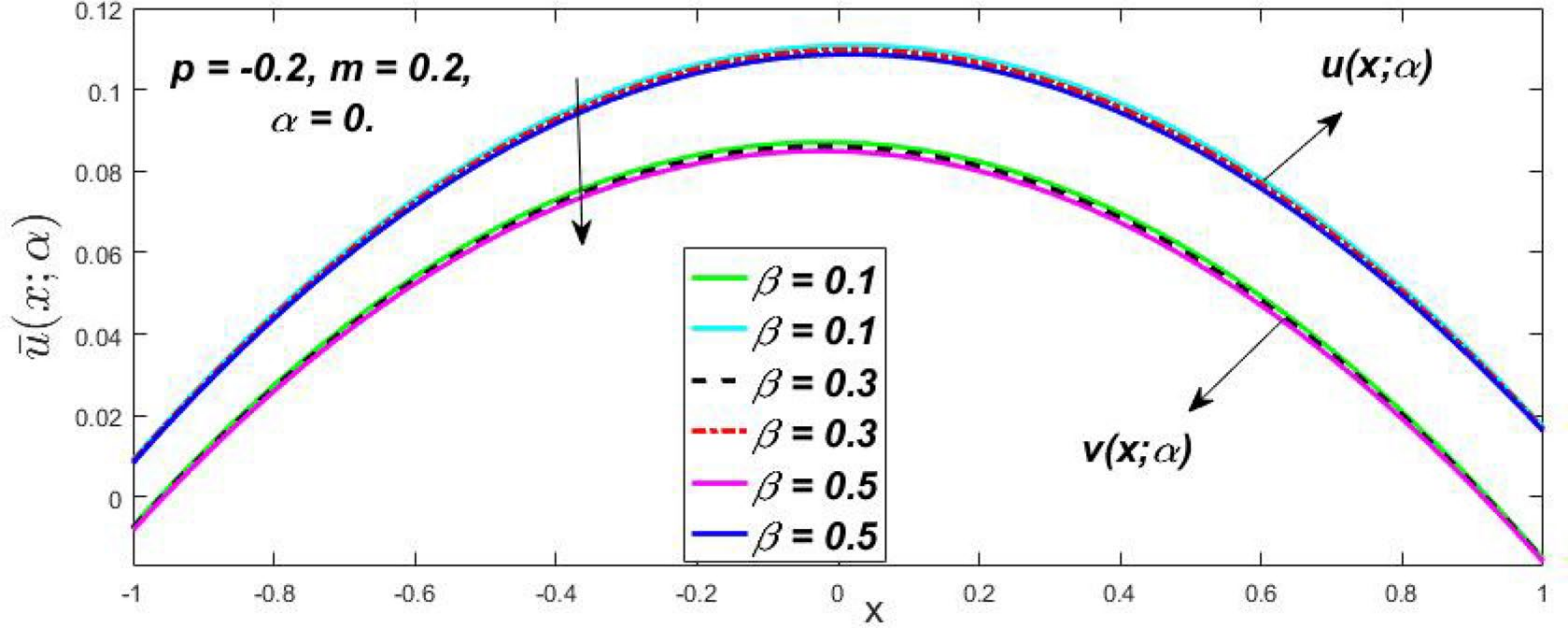
Fuzzy velocity profiles for the impact of $\beta .$

**Figure 15:**
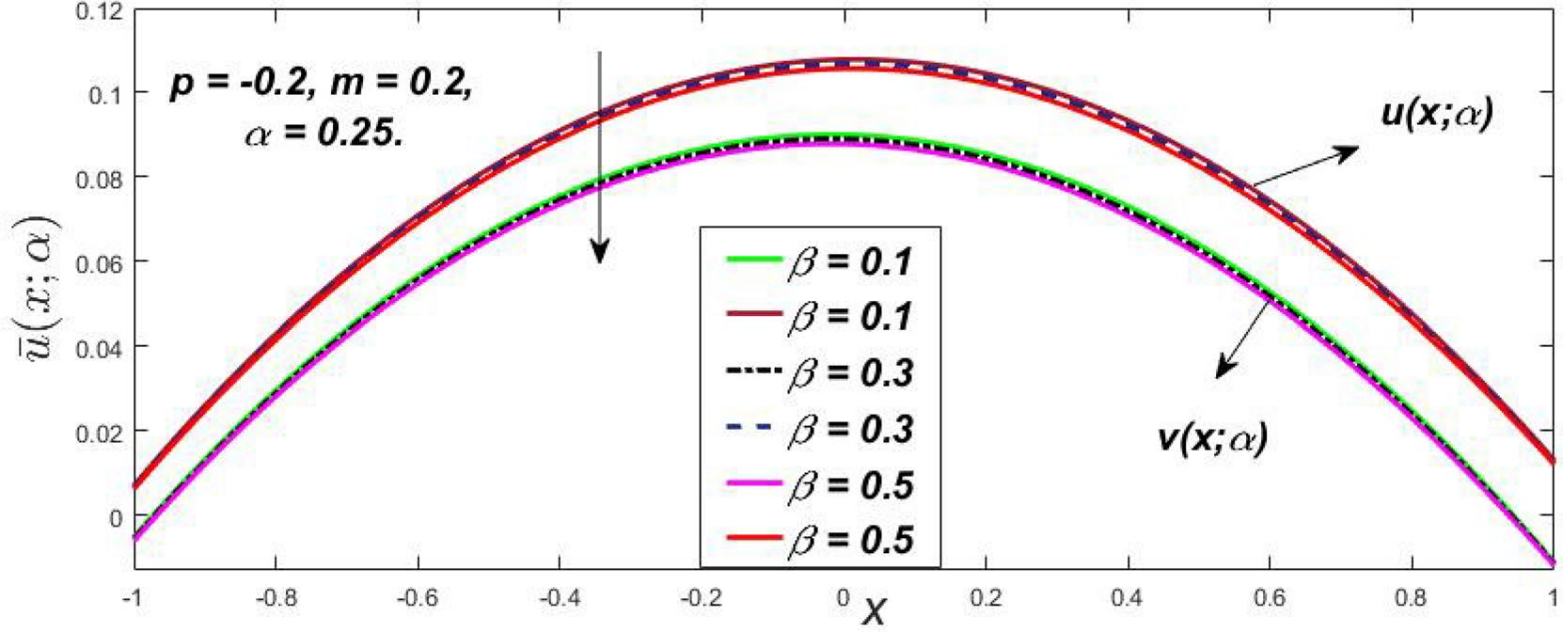
Fuzzy velocity profiles for the impact of $\beta .$

**Figure 16:**
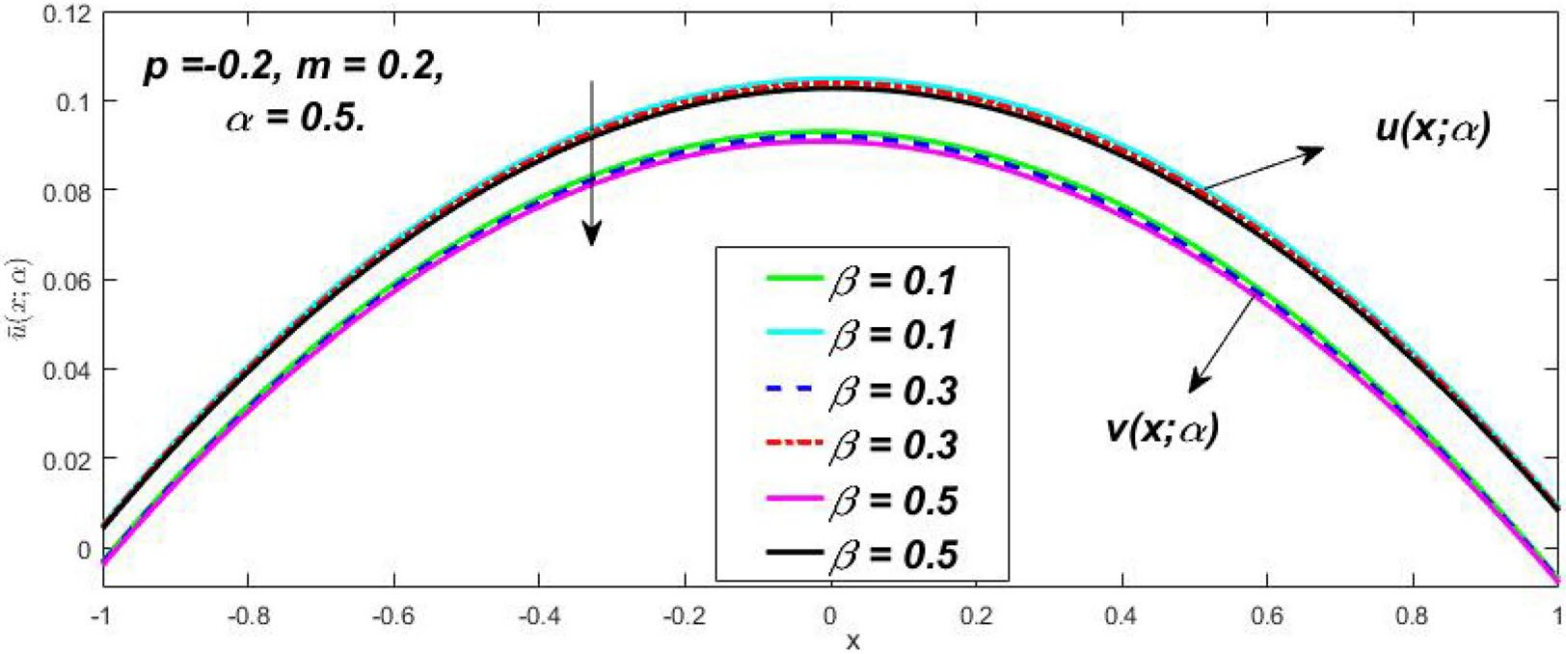
Fuzzy velocity profiles for the impact of $\beta .$

**Figure 17:**
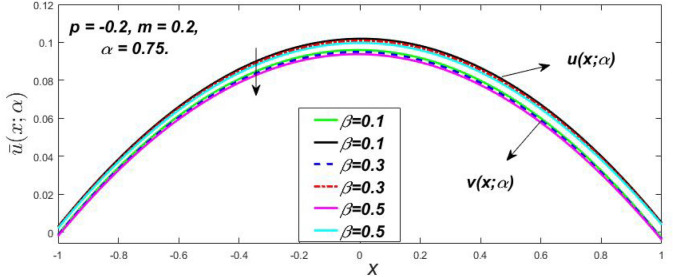
Fuzzy velocity profiles for the impact of $\beta .$

**Figure 18:**
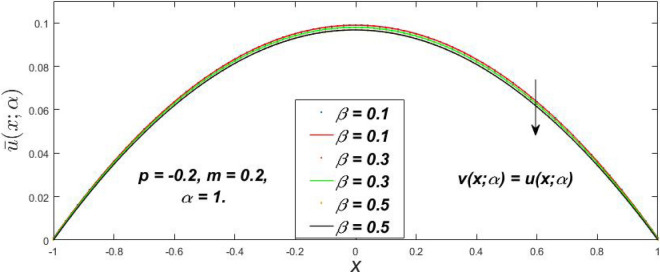
Fuzzy velocity profiles for the impact of $\beta .$

**Figure 19:**
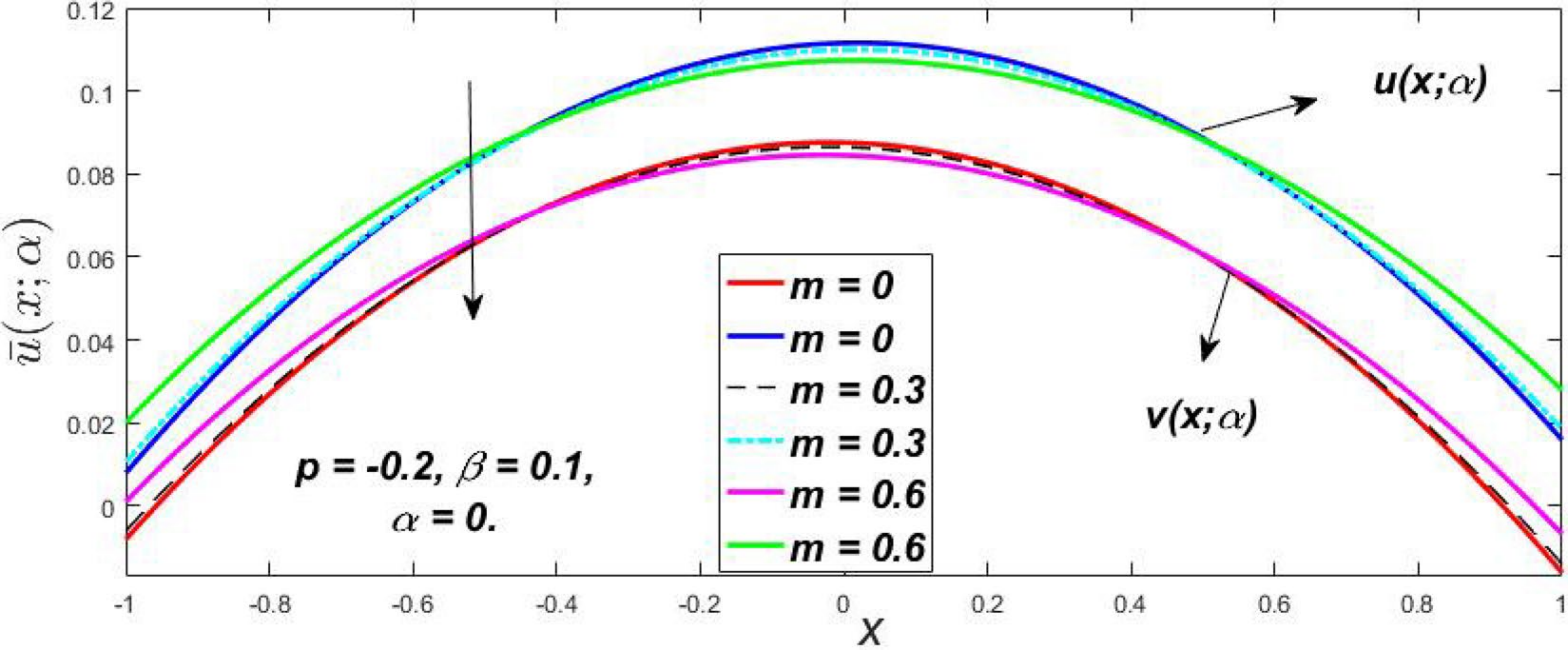
Fuzzy velocity profiles for the impact of *m.*

**Figure 20:**
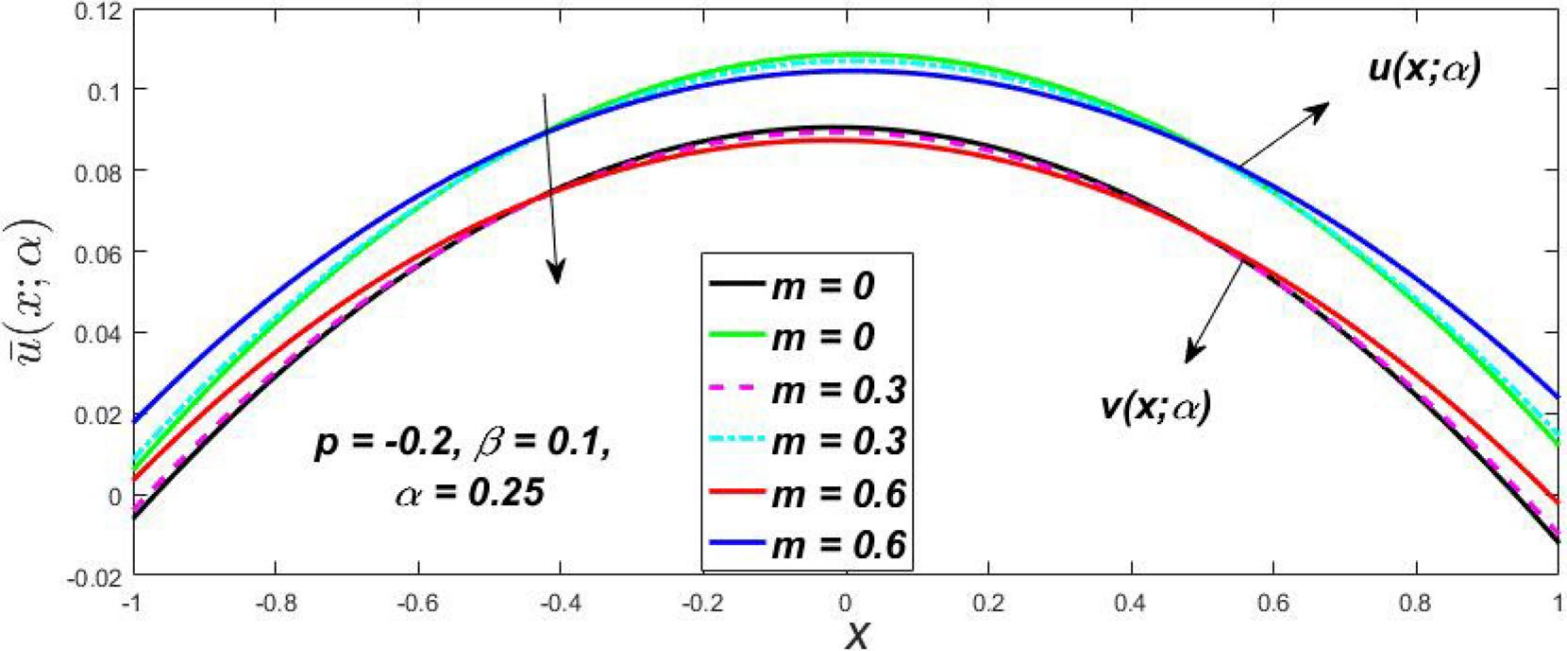
Fuzzy velocity profiles for the impact of *m.*

**Figure 21:**
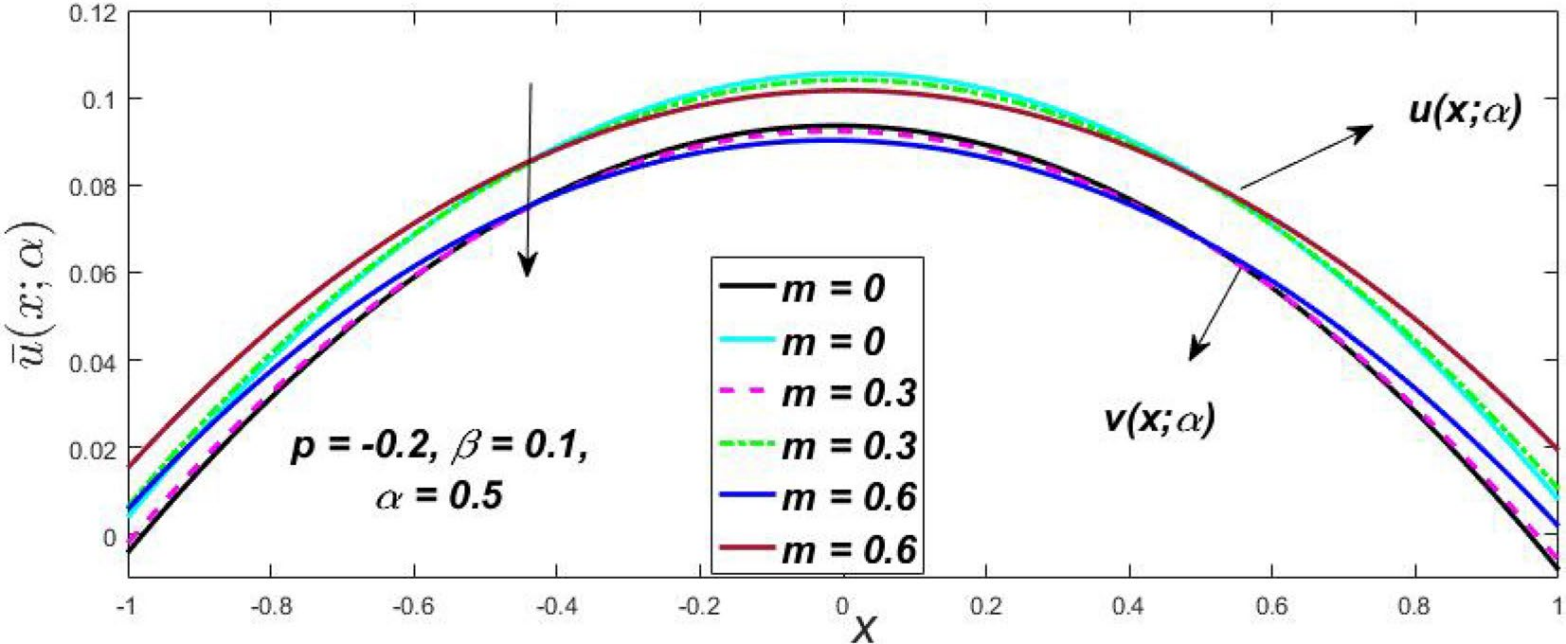
Fuzzy velocity profiles for the impact of *m.*

**Figure 22:**
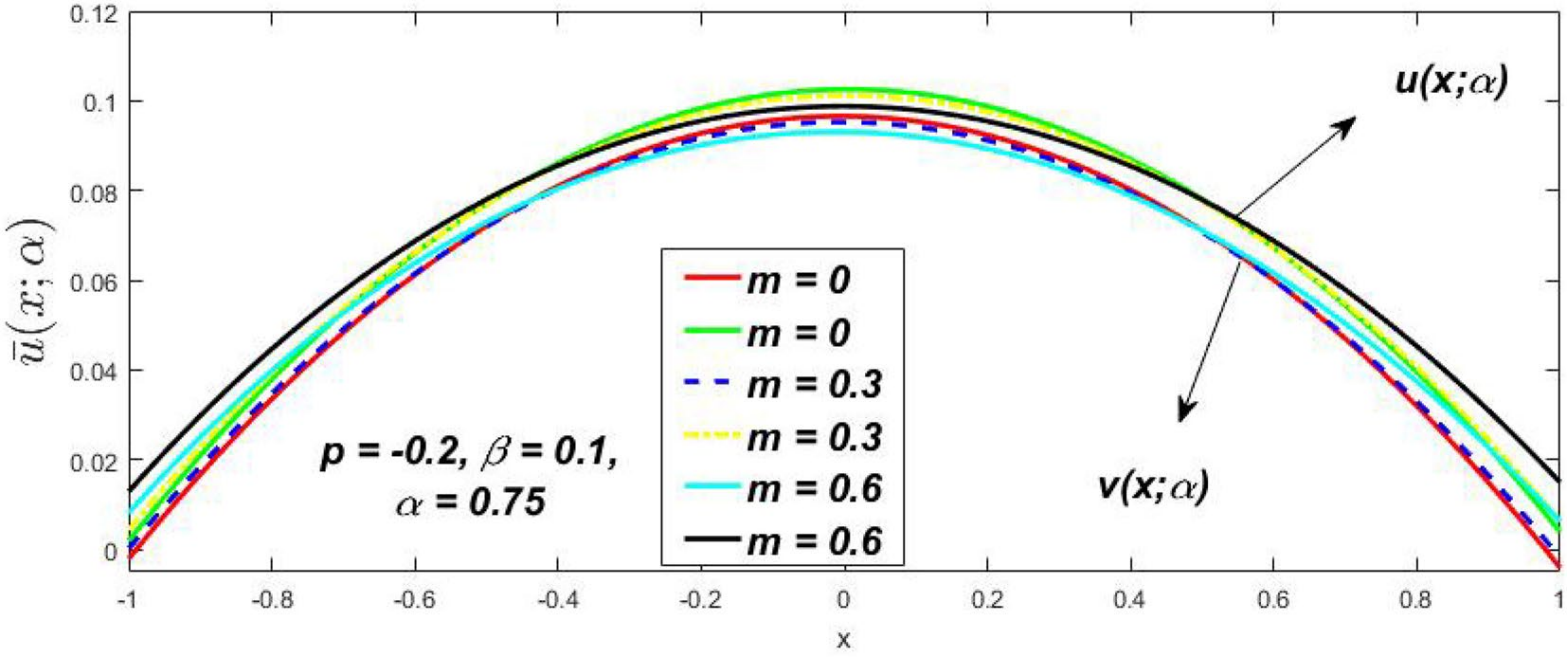
Fuzzy velocity profiles for the impact of *m.*

**Figure 23:**
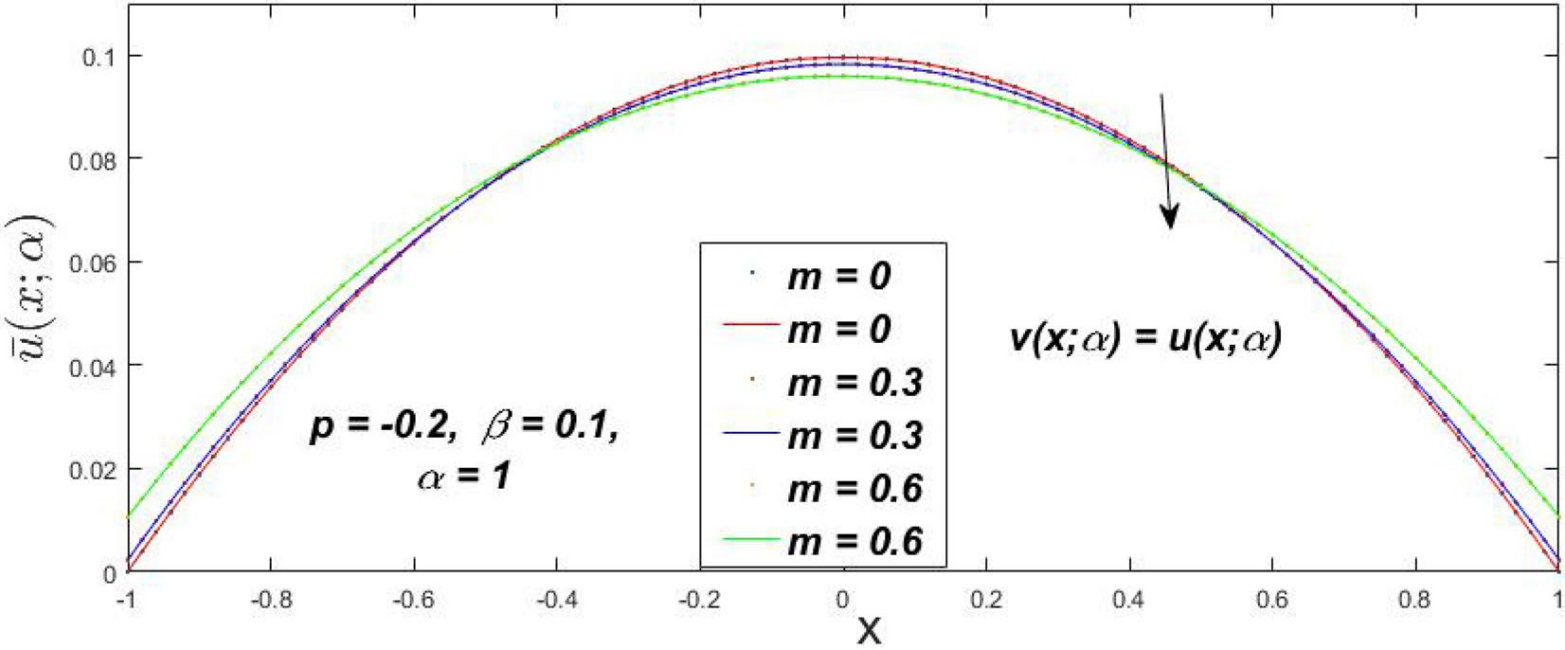
Fuzzy velocity profiles for the impact of *m.*

**Figure 24:**
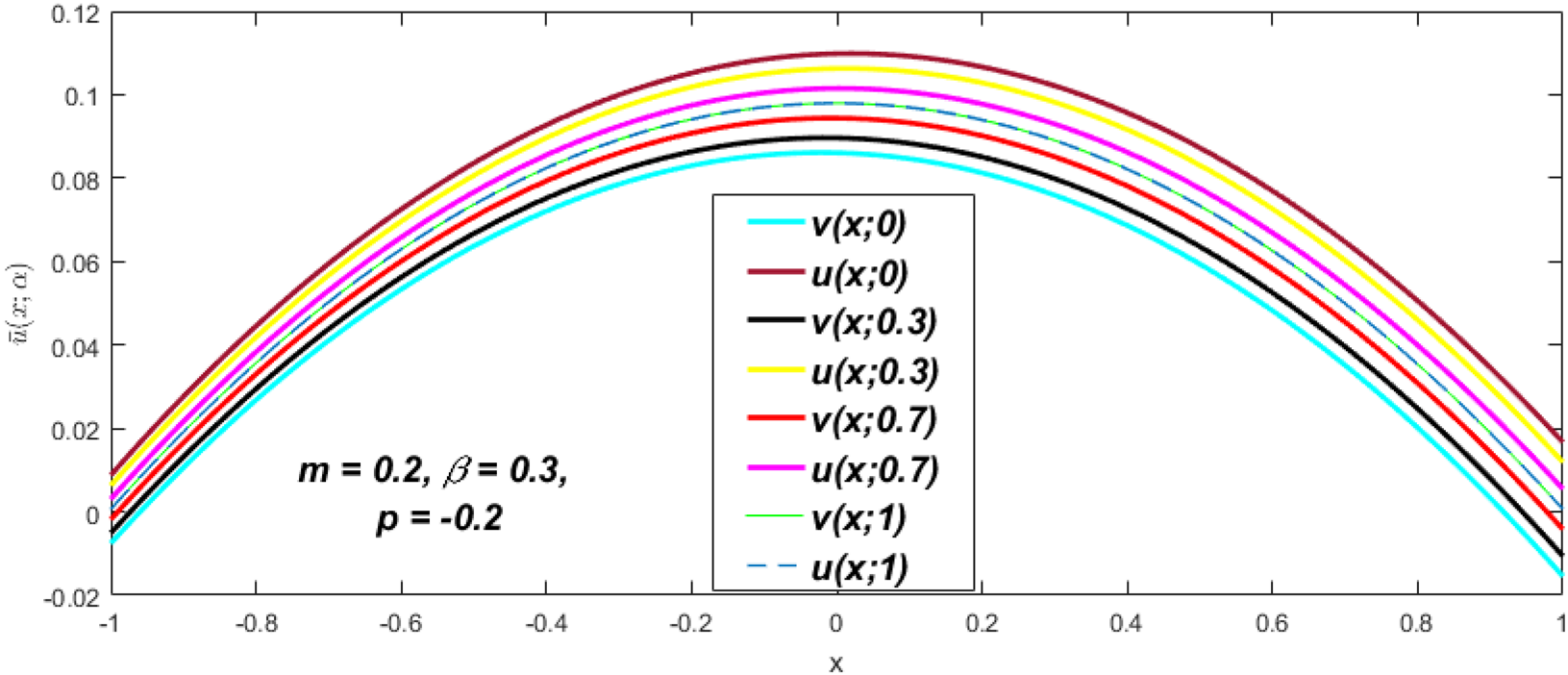
Fuzzy velocity for different values of $\alpha$-cut.

**Figure 25:**
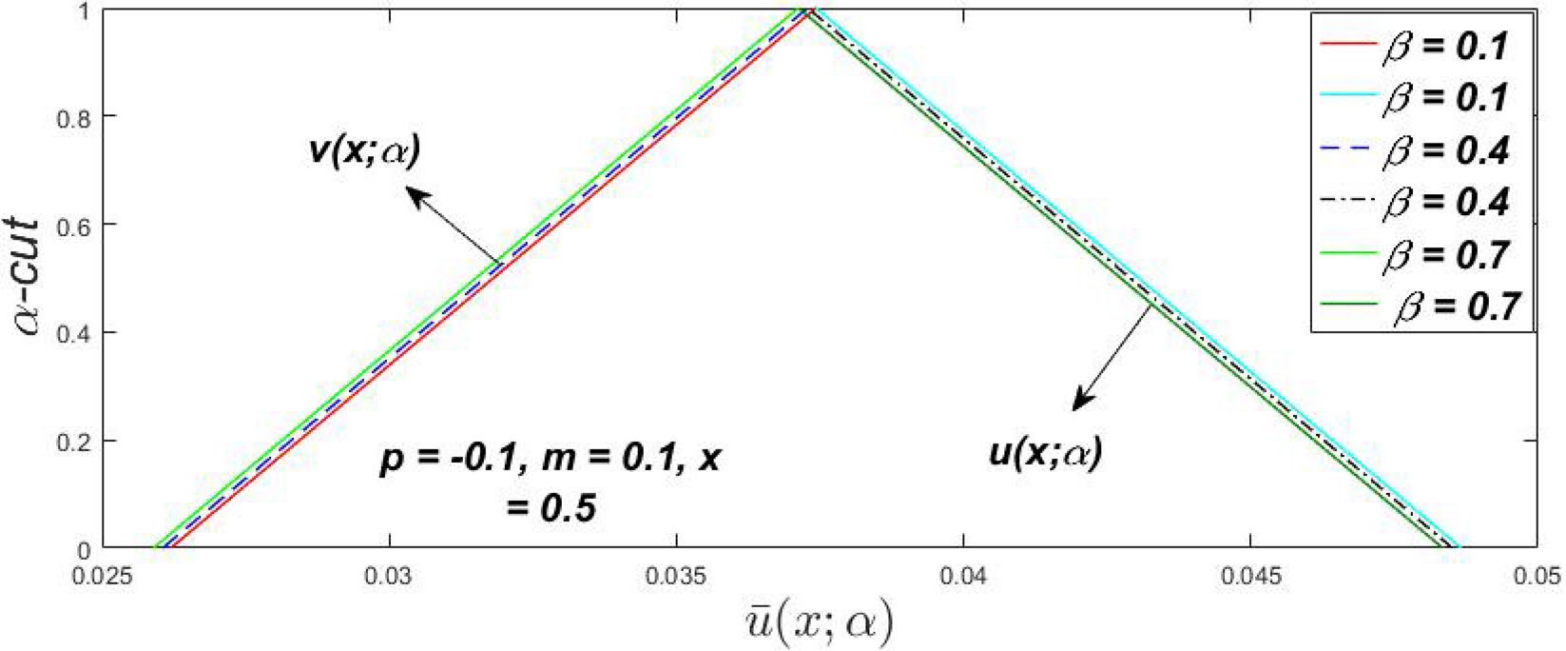
Triangular MF of fuzzy velocity for the impact of $\beta .$

**Figure 26:**
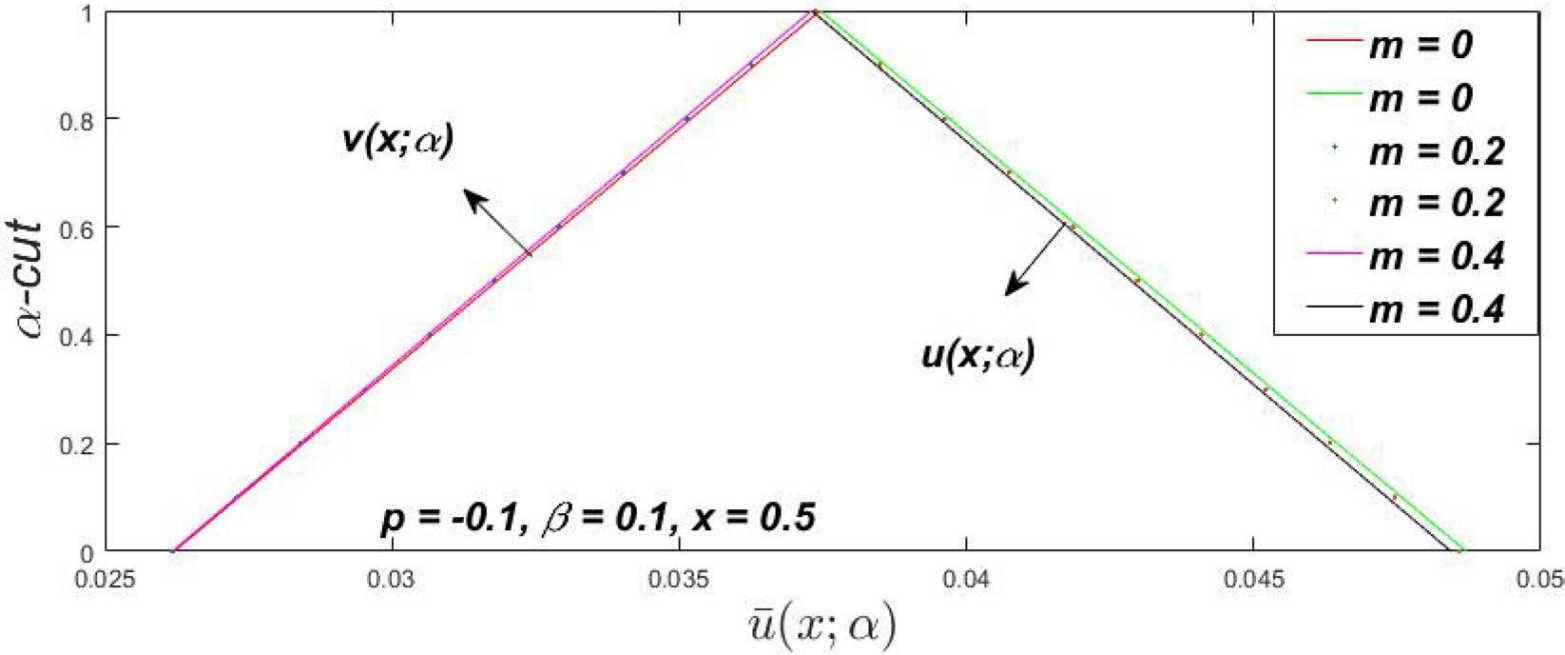
Triangular MF of fuzzy velocity for the impact of *m*.

**Figure 27:**
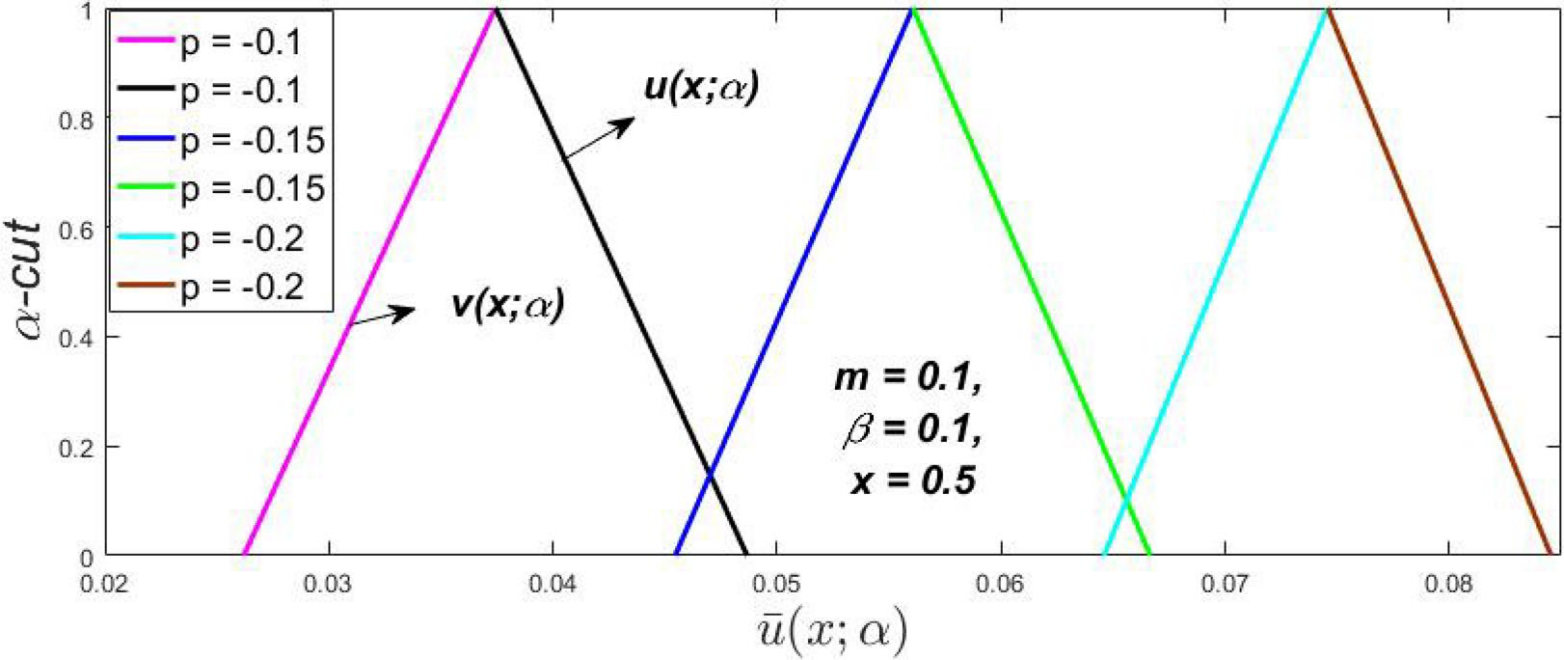
Triangular MF of fuzzy velocity for the impact of $p = {{dp} \mathord{\left/ {\vphantom {{dp} {dy}}} \right. \kern-\nulldelimiterspace} {dy}}.$

**Figure 28:**
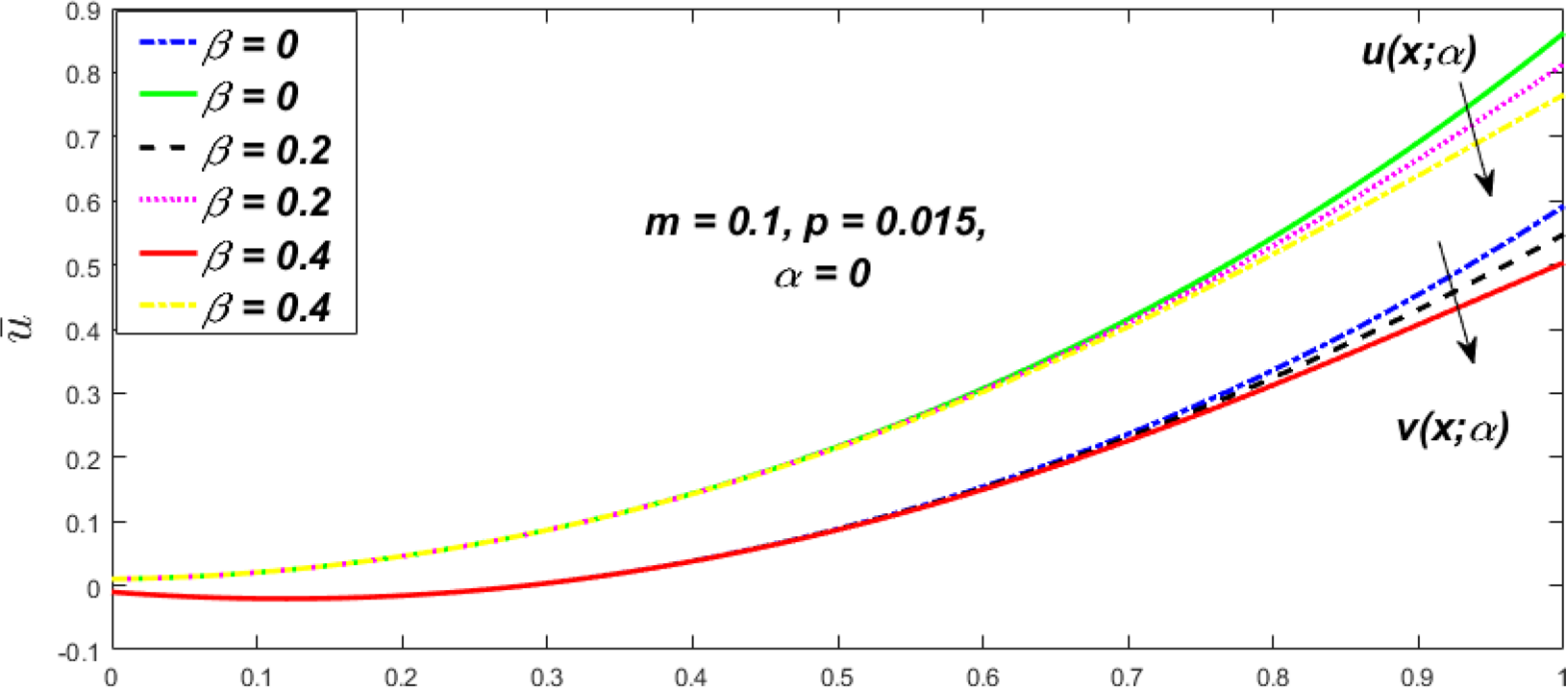
Fuzzy velocity profiles for the impact of $\beta .$

**Figure 29:**
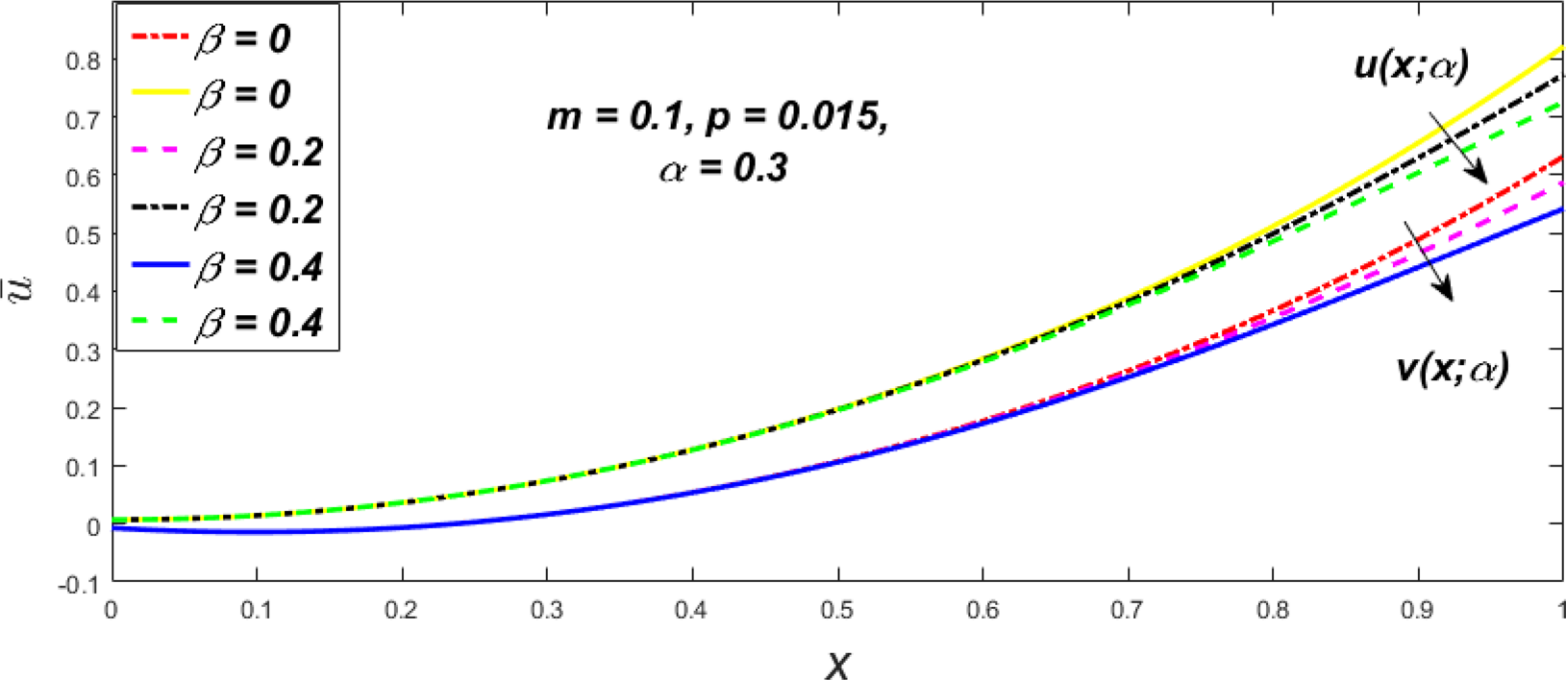
Fuzzy velocity profiles for the impact of $\beta .$

**Figure 30:**
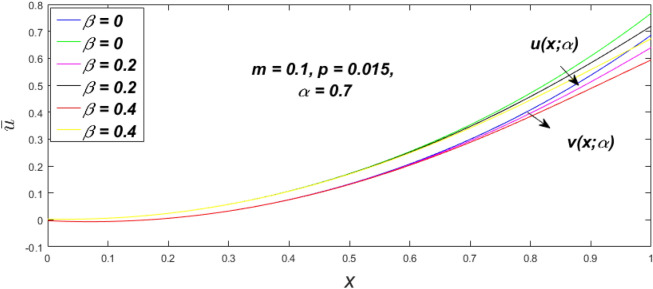
Fuzzy velocity for the impact of $\beta .$

**Figure 31:**
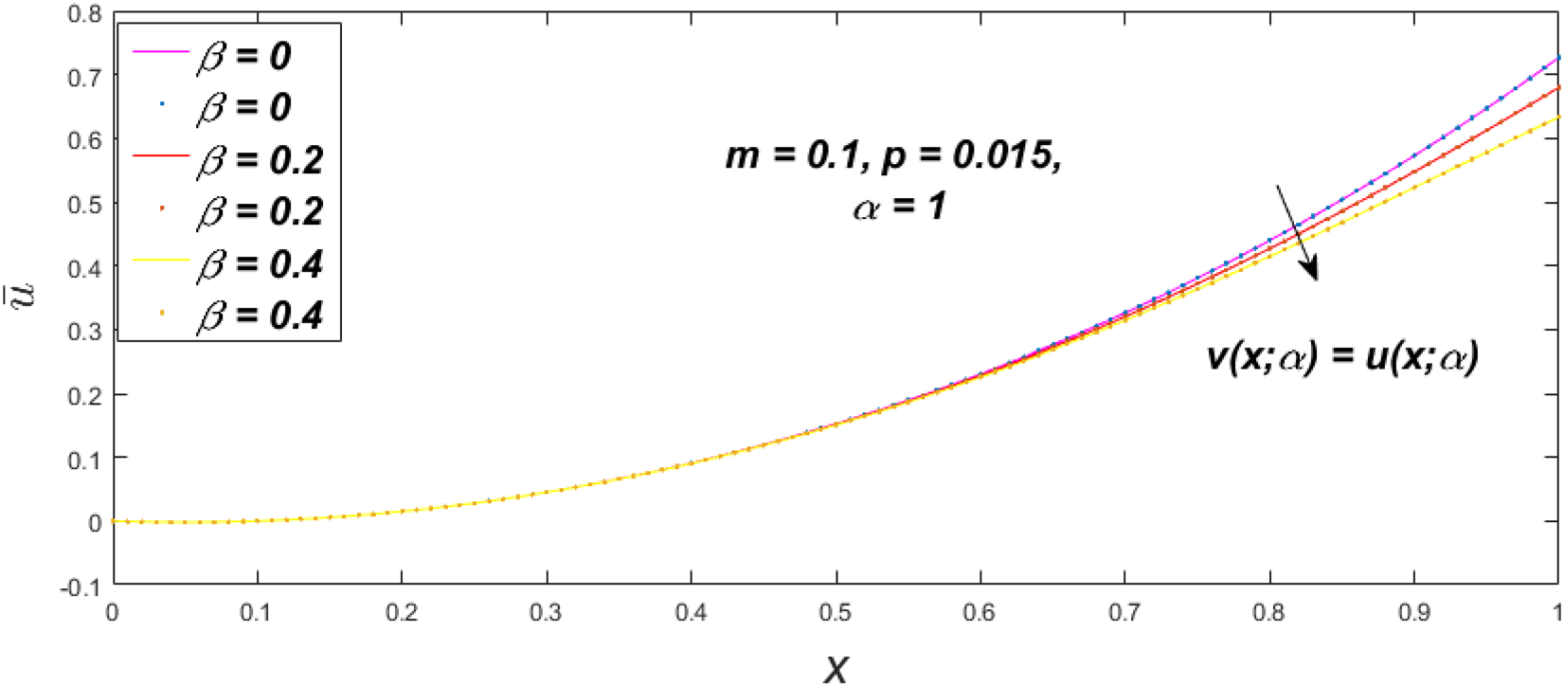
Fuzzy velocity for the impact of $\beta .$

**Figure 32:**
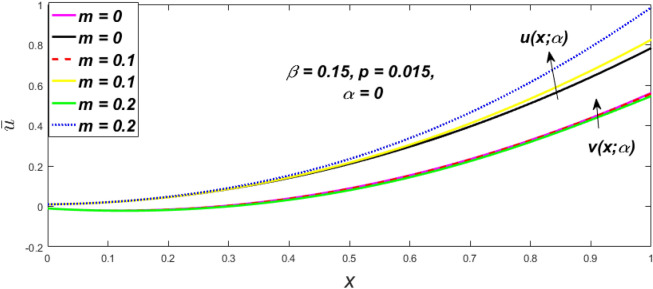
Fuzzy velocity for the impact of *m.*

**Figure 33:**
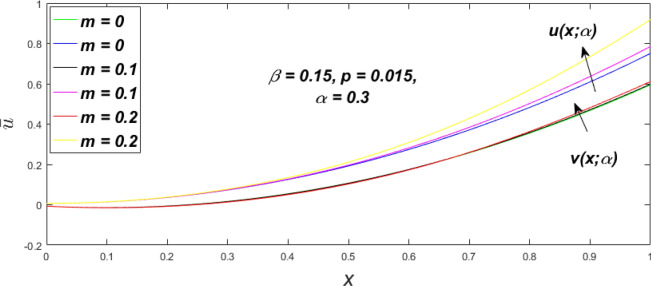
Fuzzy velocity for the impact of *m.*

**Figure 34:**
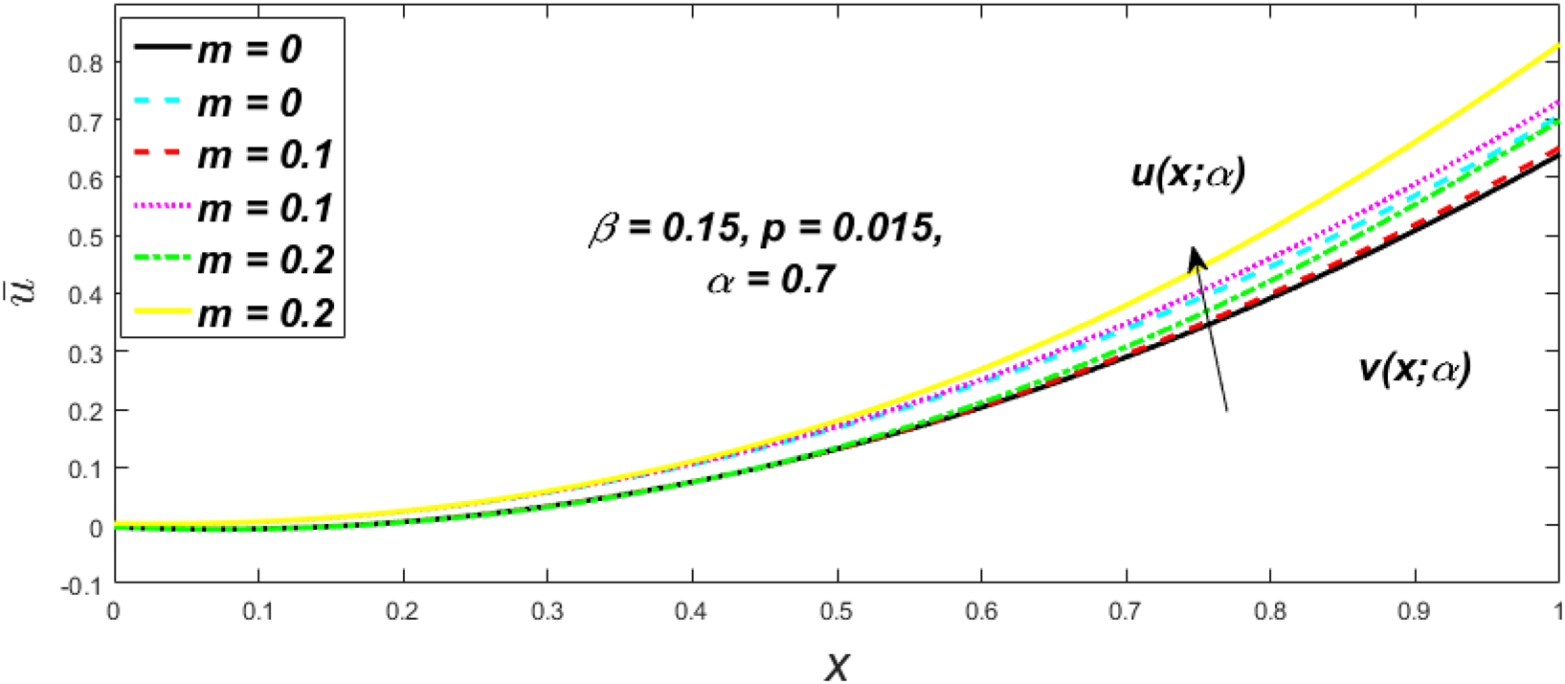
Fuzzy velocity for the impact of *m.*

**Figure 35:**
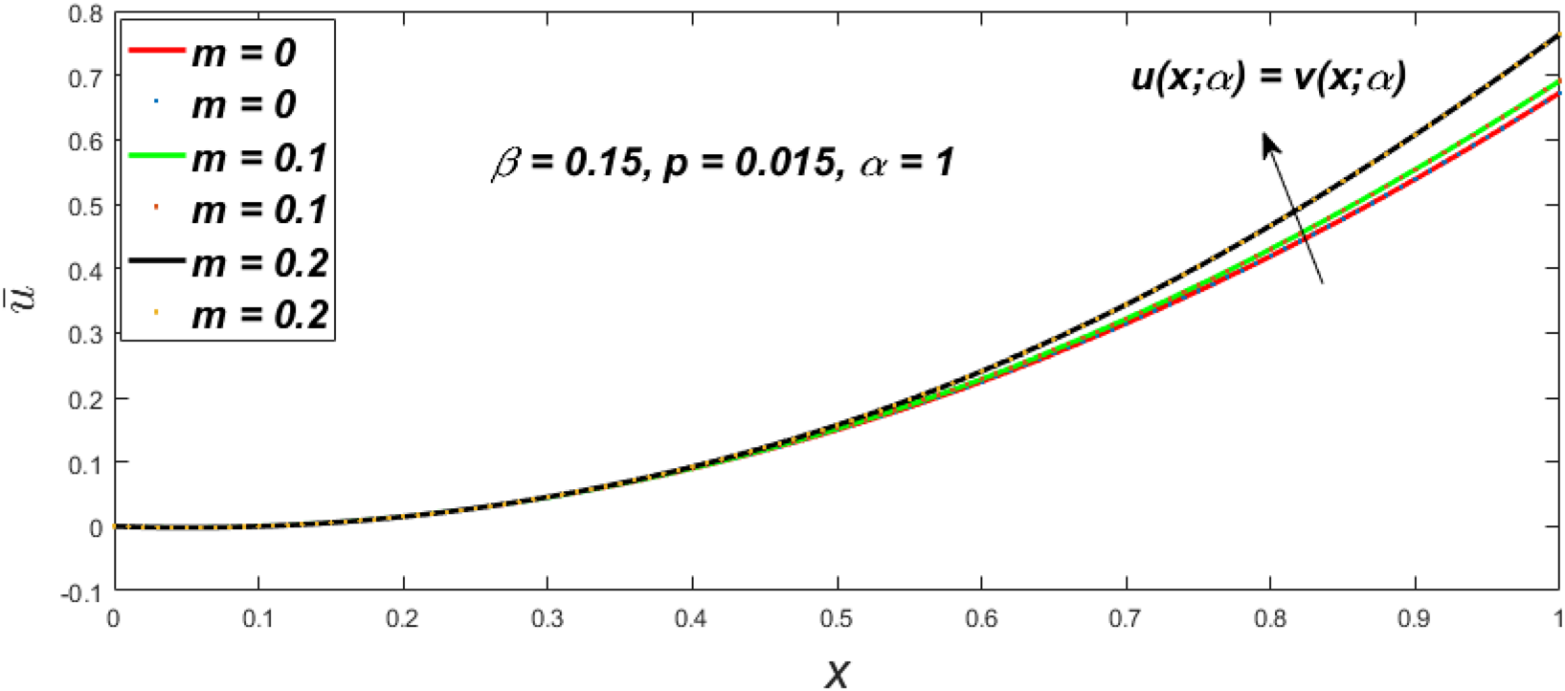
Fuzzyvelocity for the impact of *m.*

**Figure 36:**
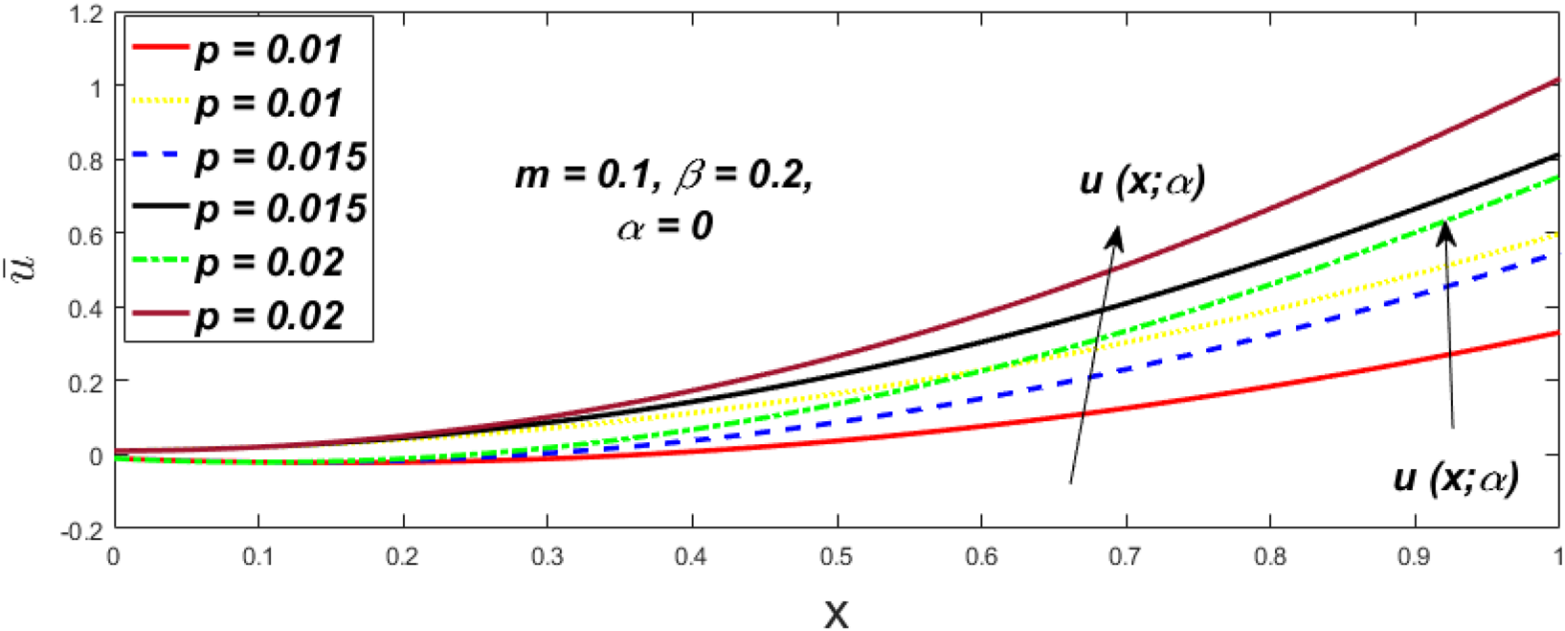
Fuzzy velocity for the impact of *p.*

**Figure 37:**
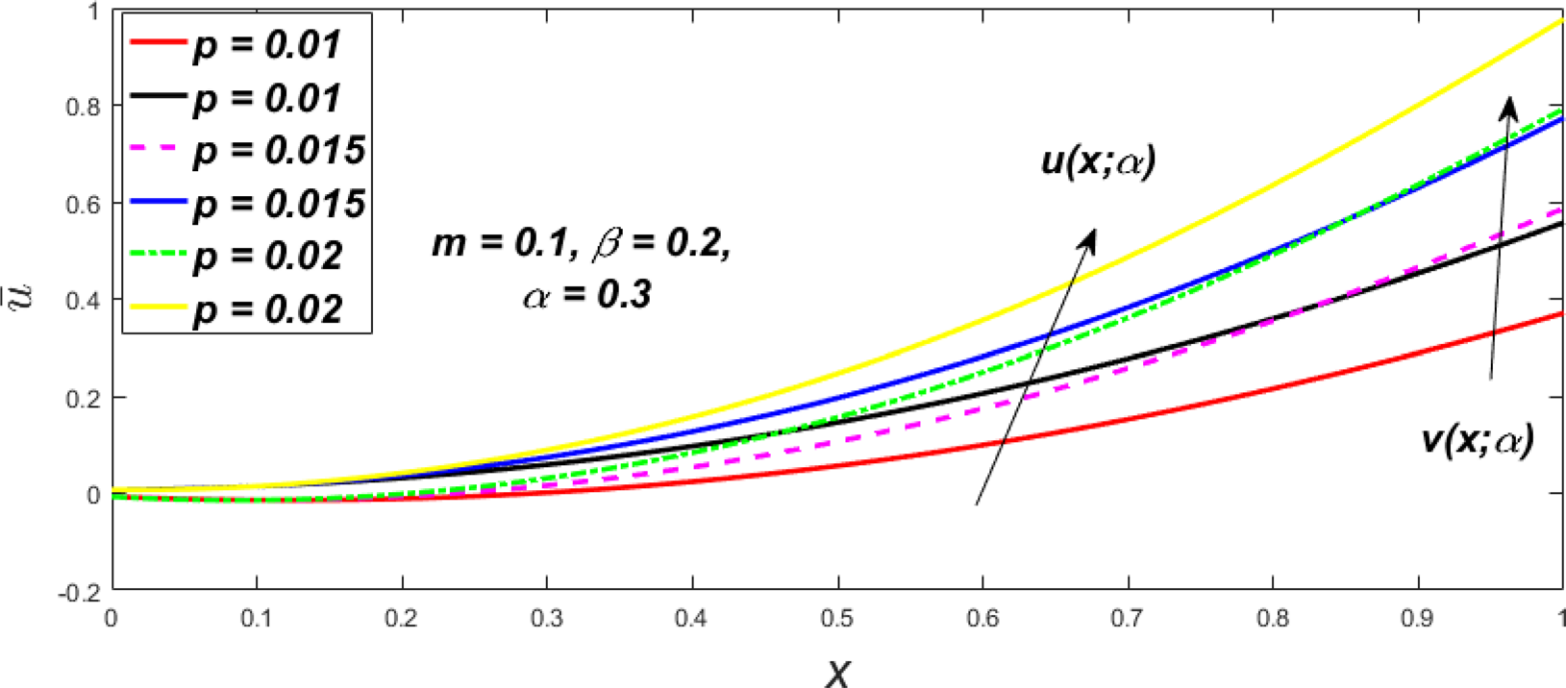
Fuzzy velocity for the impact of *p.*

**Figure 38:**
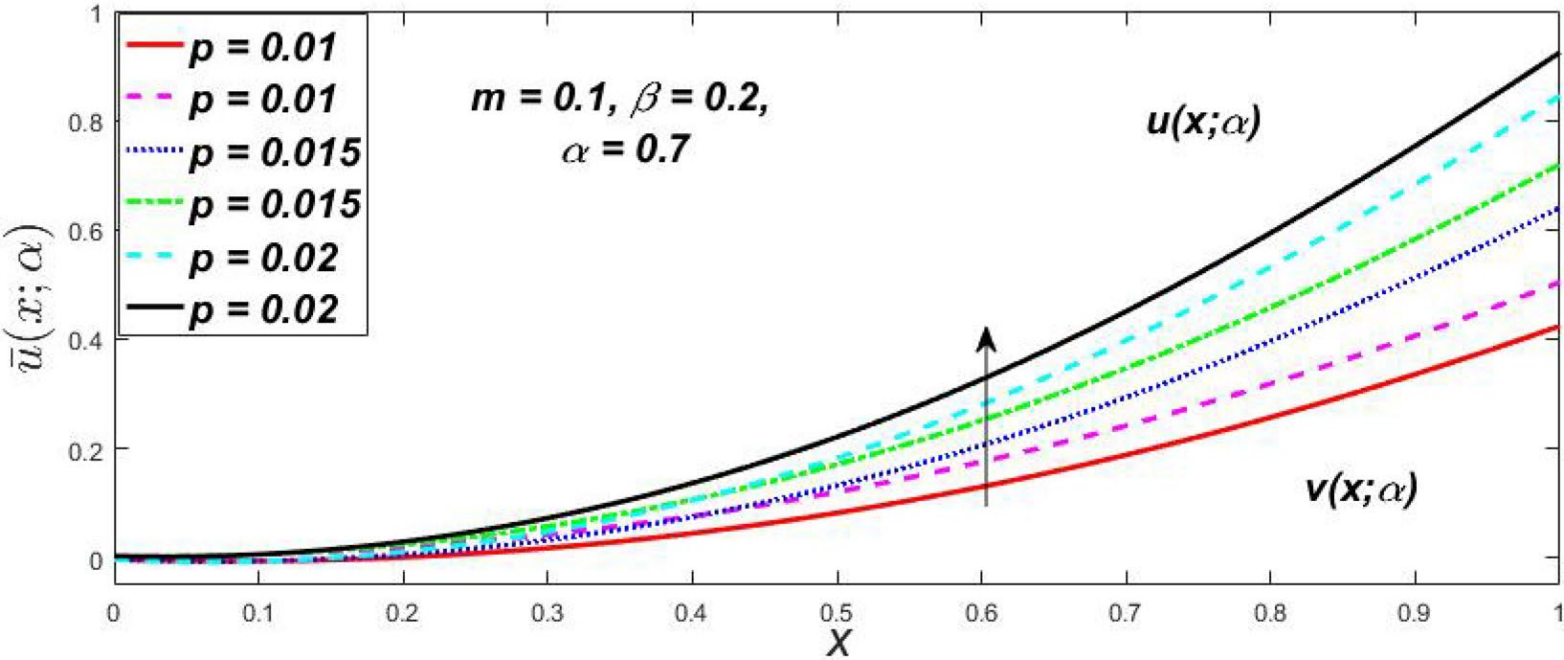
Fuzzy velocity for the impact of *p.*

**Figure 39:**
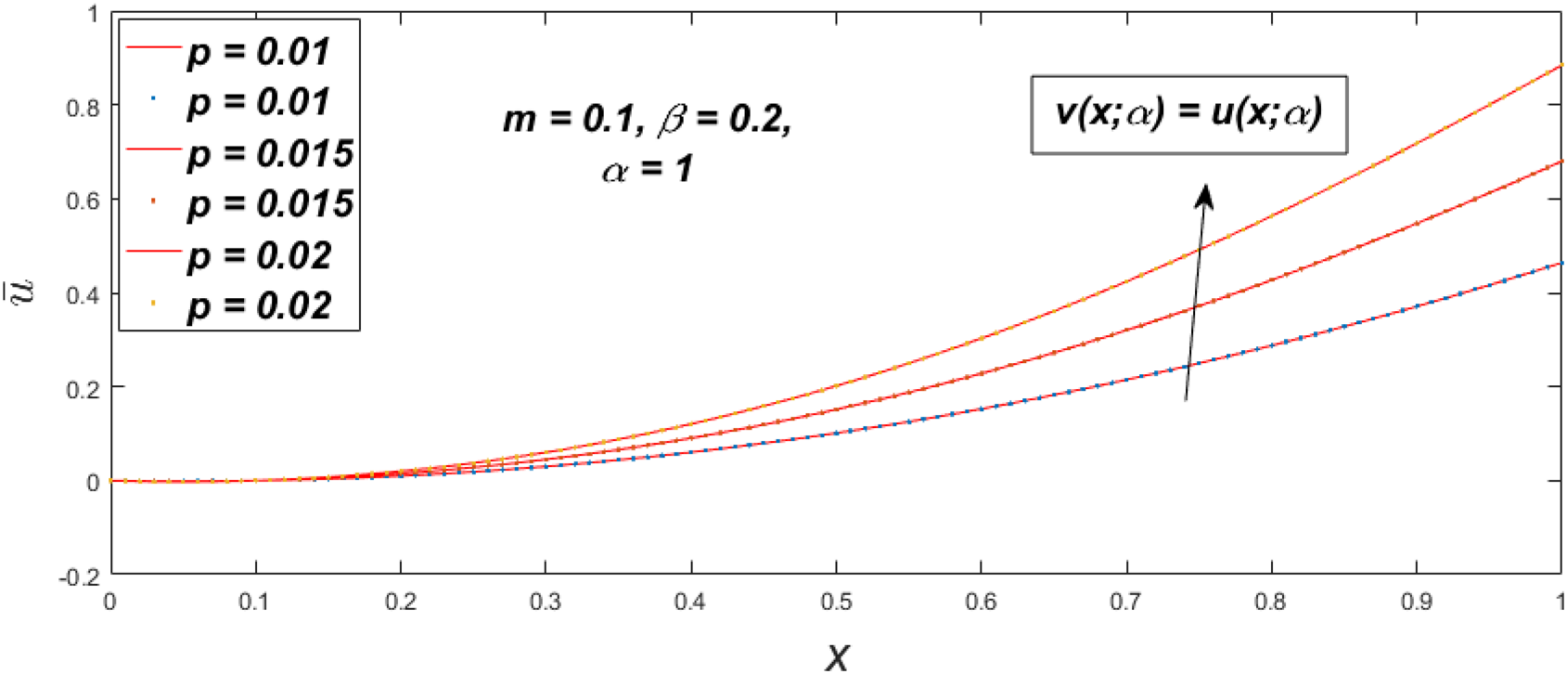
Fuzzy velocity for the impact of *p.*

**Figure 40:**
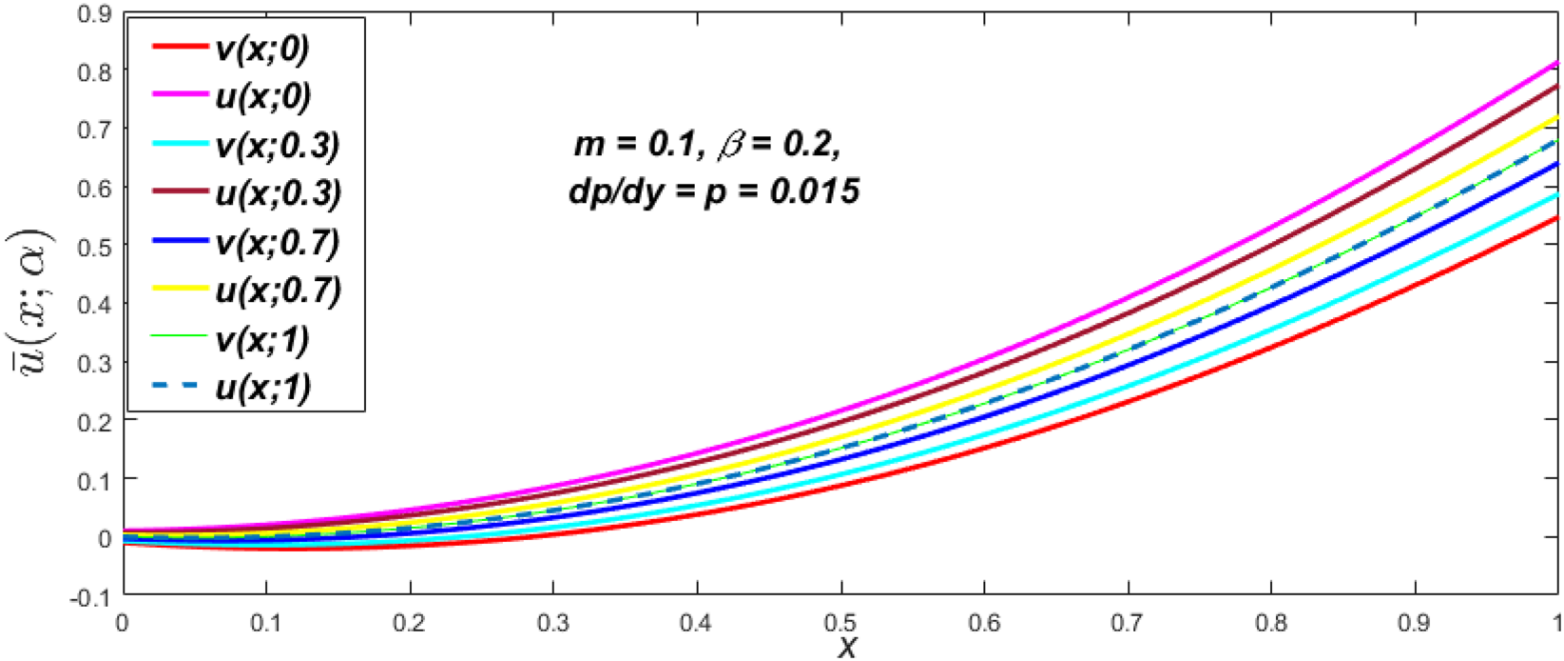
Fuzzy velocity for numerous values of $\alpha$-cut.

**Figure 41:**
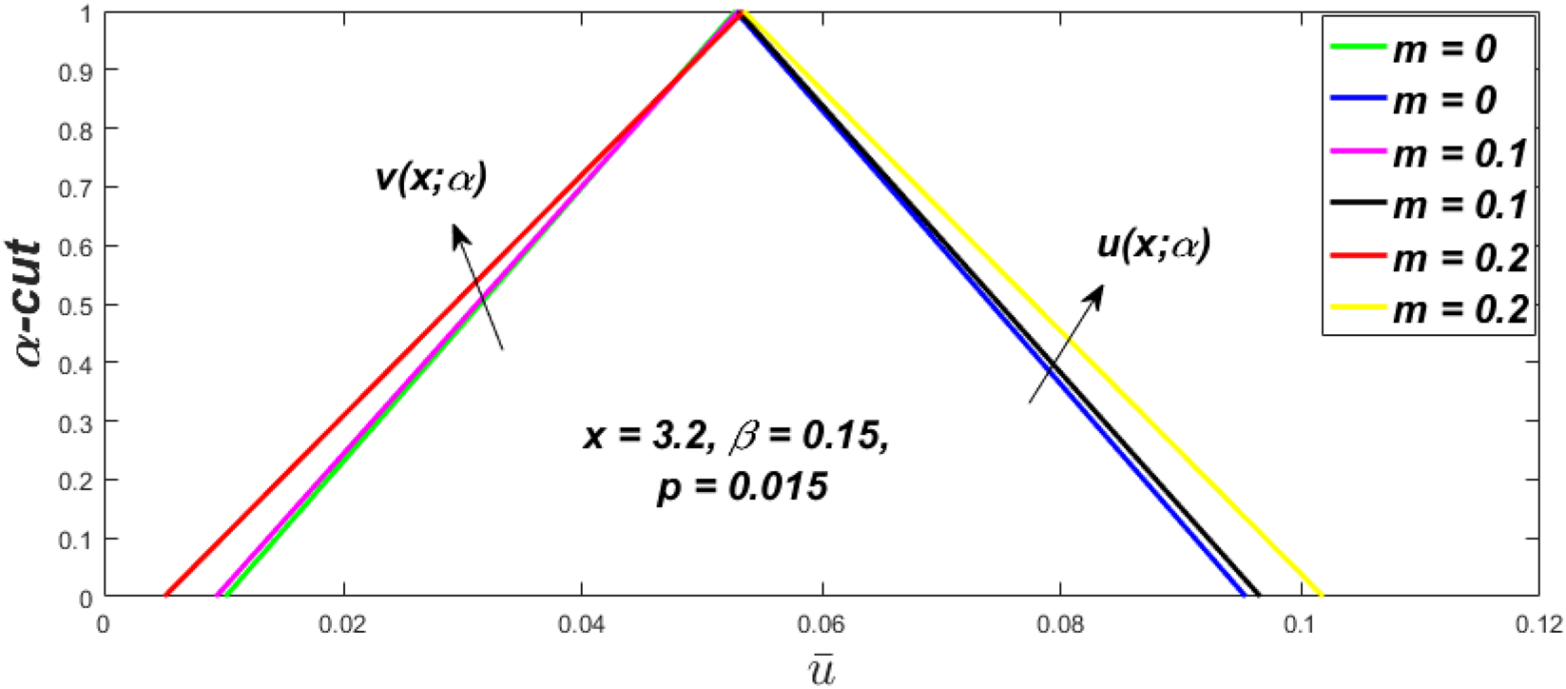
Triangular MF of the fuzzy velocity for the impact of *m.*

**Figure 42:**
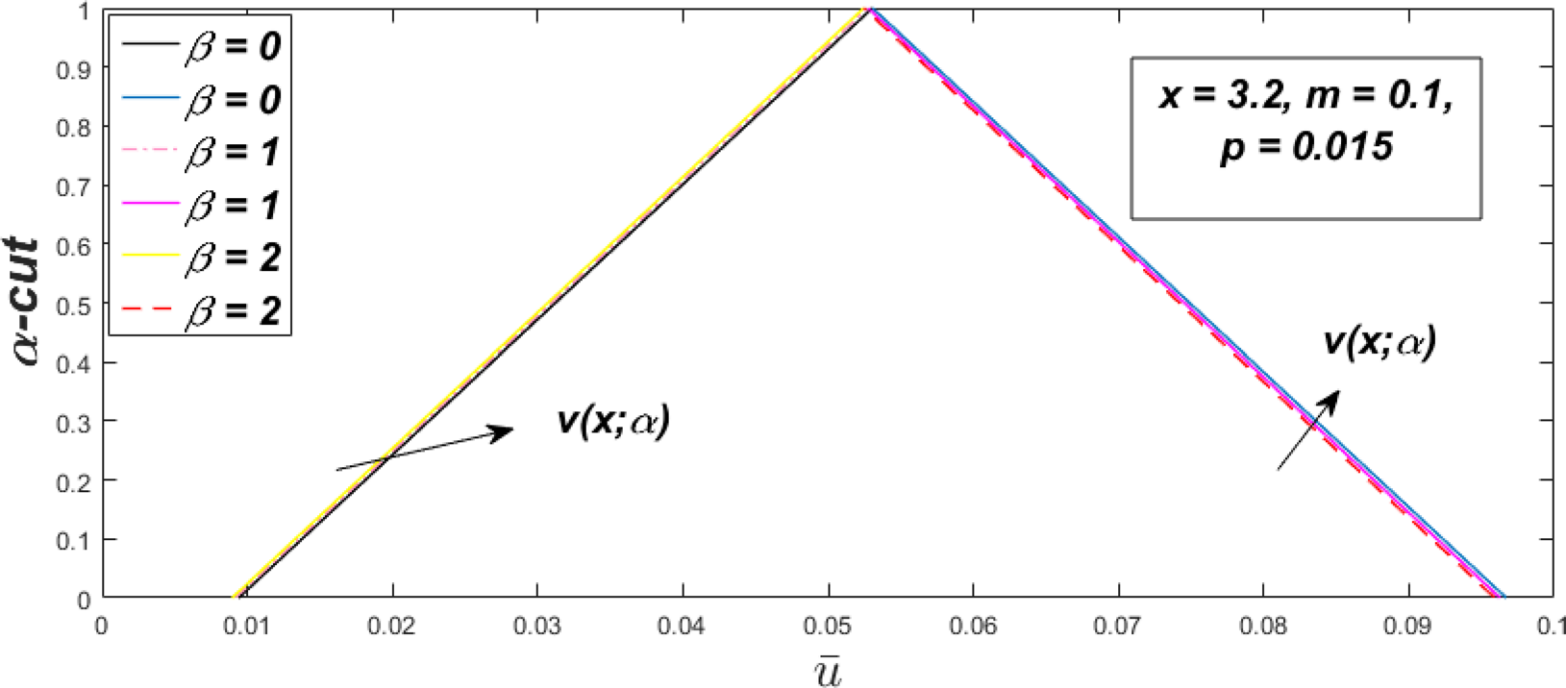
Triangular MF of fuzzy velocity for different values of $\beta .$

**Figure 43:**
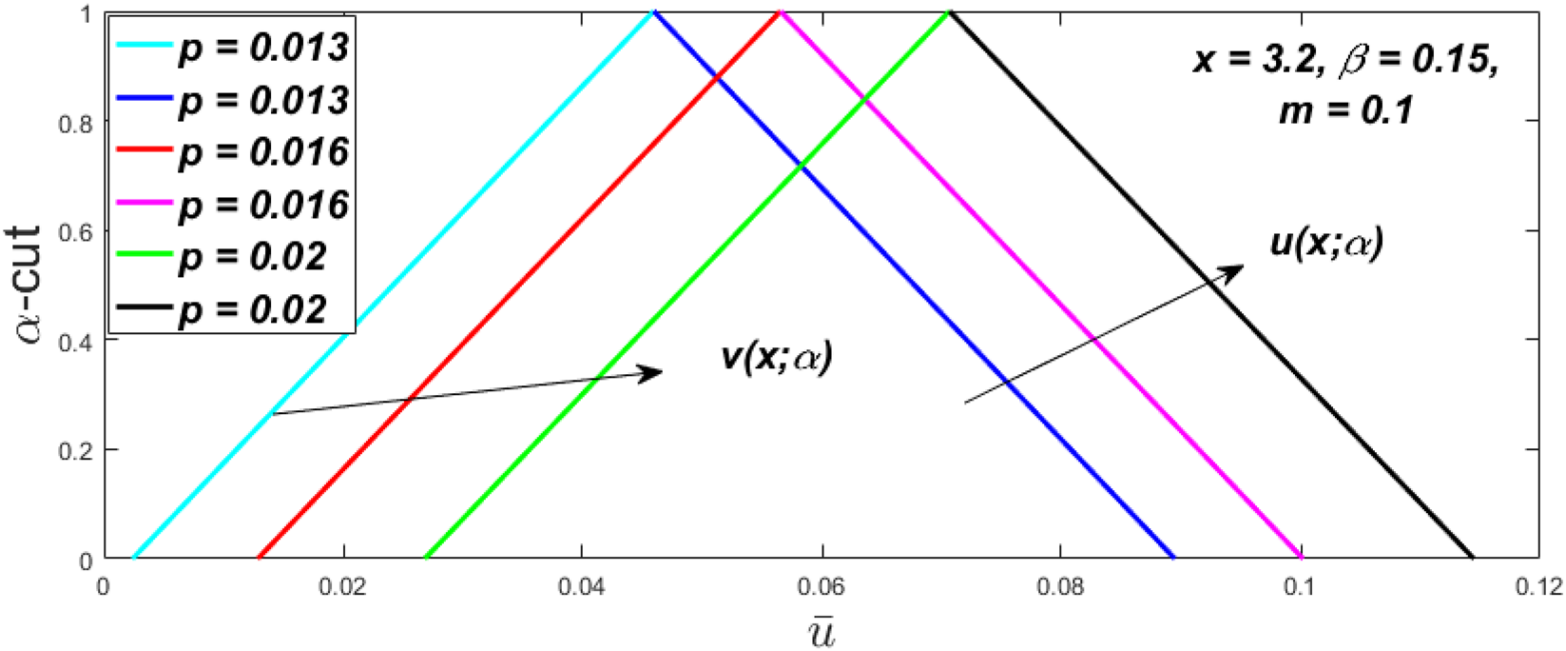
Triangular MF of fuzzy velocity for the impact of *p.*

Tables 1, 2 and 3 show the comparison of the crisp velocity profile with Siddiqui et al.^4^ and Yürüsoy^9^. The validation of the present study findings was determined to be in excellent agreement.

**Table 1:** Comparison of analytical results for the crisp velocity profile of Couette flow when $\beta = 0.1,\,\,m = 0,\,\,p = - 0.5\,\,{\text{and}}\,\,\alpha {\text{-cut}} = 1.$

x	Siddiqui et al.^4^	Yürüsoy et al.^9^	Kamran and Siddique^14^	ADM present results
0	0	0	0	0
0.1	0.0814178	0.091211	0.076018	0.076017
0.2	0.1618119	0.181128	0.151479	0.151478
0.3	0.2618251	0.301241	0.249147	0.249145
0.4	0.3410156	0.374915	0.330156	0.330151
0.5	0.4721819	0.467189	0.476186	0.476182
0.6	0.5215171	0.572820	0.518252	0.518251
0.7	0.6214168	0.667189	0.610428	0.610425
0.8	0.7514268	0.771810	0.741514	0.741516
0.9	0.8810148	0.882185	0.871813	0.871816
1	1	1	1	1

**Table 2:** Comparison of analytical results for the crisp velocity profile of Poiseuille flow when $\beta = 0.1,\,\,m = 0,\,\,\,p = - 0.5\,\,{\text{and}}\,\,\alpha {\text{-cut}} = 1.$

x	Siddiqui et al.^4^	Yürüsoy et al.^9^	Kamran and Siddique^14^	ADM present results
0	0.049141	0.049792	0.048813	0.048812
0.1	0.048818	0.049211	0.048315	0.048314
0.2	0.046718	0.047801	0.045145	0.045140
0.3	0.045168	0.045314	0.044143	0.044141
0.4	0.041015	0.041830	0.040018	0.040019
0.5	0.037180	0.037351	0.036126	0.036124
0.6	0.031417	0.031876	0.031103	0.031109
0.7	0.025001	0.025404	0.024916	0.024914
0.8	0.017912	0.017934	0.017815	0.017819
0.9	0.009214	0.009466	0.009167	0.009164
1	0	0	0	0

**Table 3:** Comparison of analytical results for the crisp velocity profile of Couette–Poiseuille flow when $\beta = 0.1,\,m = 0,\,\,\,p = - 0.5\,\,{\text{and}}\,\,\alpha {\text{-cut}} = 1.$

x	Siddiqui et al.^4^	Yürüsoy et al.^9^	Kamran and Siddique^14^	ADM present results
0	0	0	0	0
0.1	0.081018	0.091401	0.044183	0.044181
0.2	0.121481	0.020762	0.109166	0.109168
0.3	0.194415	0.300141	0.191622	0.191620
0.4	0.281510	0.411359	0.266406	0.266405
0.5	0.380151	0.521415	0.361792	0.361791
0.6	0.481141	0.591618	0.471814	0.471819
0.7	0.601486	0.691619	0.599162	0.599160
0.8	0.721412	0.791819	0.714993	0.714991
0.9	0.881514	0.900410	0.871680	0.871680
1	1	1	1	1

### Plane Couette flow

At distinct values of $\alpha {\text{-cut}}$ $\left( {0 \le \alpha \le 1} \right),$ the upshot of a magnetic parameter (*m*) on the $v(x,\,\alpha )$ and $u(x,\,\alpha )$ with non-Newtonian fluid, parameters $\left( \beta \right)$ is exposed in Figs. 3, 4, 5 and 6. For various values of $\alpha {\text{-cut,}}$ the crisp velocity profile is generalised so the $v(x,\,\alpha )$ and $u(x,\,\alpha )$ reduce gradually near the centre of the plates as the *m* upsurges. The influence of $\beta$ on the $v(x,\,\alpha )$ and $u(x,\,\alpha )$ for *m* at varying values of the $\alpha {\text{-cut}}$ is shown in Figs. 7, 8, 9 and 10. At different values of $\alpha ,$ the fuzzy flow rates grow gradually near the middle of the plates with rising $\beta .$ It has a favourable influence in Figs. 6 and 10 to give a classic solution in which the $v(x,\,\alpha )$ and $u(x,\,\alpha )$ are the same at $\alpha = 1.$ Figure 11 shows the $v(x,\,\alpha )$ and $u(x,\,\alpha )$ for various $\alpha$ values. Because the crisp velocity profile lies between the $v(x,\,\alpha )$ and $u(x,\,\alpha )$, the fuzzy velocity drops into the crisp velocity profile when $\alpha = 1,$ indicating that the current problem is a expansion of Kamran and Siddique^14^. Figures 12 and 13 depict the uncertain response of the TFN memberships function with the triangle fuzzy plot when $\beta$ and *m* are varied. Figure 12 shows the fuzzy width declines through growing input *m*, but Fig. 13 demonstrates how the uncertain width grows with rising $\beta .$ It was also discovered that when $\alpha$ grows, the $v(x,\,\alpha )$ upsurges and the $u(x,\,\alpha )$ drops, implying that the solutions are powerful. The width between the $v(x,\,\alpha )$ and $u(x,\,\alpha )$ narrows as $\alpha$ grows, and at $\alpha = 1,$ they coherent with the origenal answer. The analysis of $v(x,\,\alpha )$, mid, and $u(x,\,\alpha )$ for various values of *x* with $\beta = 0.75\,\,{\text{and}}\,\,m = 1$ are presented in Table 4. The crisp value of the original problem agrees with the TFN's mid-value. Furthermore, for every set $\alpha$-cut, fuzzy velocity profiles always change within a particular range, and the range steadily diminishes as the $\alpha$-cut values improve.

**Table 4:** Fuzzy solution of $v(x,\,\alpha )$, mid and $u(x,\,\alpha )$ at $\,\,m = 1,\,\,\alpha {\text{-cut}} = 0,\,\,{\text{and}}\,\,\beta = 0.75$ with varing of *x*.

x	$v\left( {x;\,\alpha } \right)$	Mid values	$u\left( {x;\,\alpha } \right)$
0	− 0.01000000	0	0.01000000
0.1	− 0.00229948	0.00807392	0.01844732
0.2	0.00537698	0.01622165	0.02706633
0.3	0.01310203	0.02451870	0.03593537
0.4	0.02094899	0.03304242	0.04513585
0.5	0.02899275	0.04187300	0.05475324
0.6	0.03731059	0.05109436	0.06487813
0.7	0.04598305	0.06079512	0.07560720
0.8	0.05509474	0.07106953	0.08704432
0.9	0.06473526	0.08201839	0.09930151
1	0.07500000	0.093750000	0.11250000

### Plane Poiseuille flow

At varying values of $\alpha ,$ the impact of $\beta$ on the $v(x,\,\alpha )$ and $u(x,\,\alpha )$ with relentless pressure gradient $\left( {{{dp} \mathord{\left/ {\vphantom {{dp} {dy}}} \right. \kern-\nulldelimiterspace} {dy}} = p} \right)$ was seen in Figs. 14, 15, 16, 17 and 18. With rising $\beta ,$ the $v(x,\,\alpha )$ and $u(x,\,\alpha )$ fall. At various values of $\alpha ,$ the influence of *m* on the $v(x,\,\alpha )$ and $u(x,\,\alpha )$ with constant pressure, a gradient $\left( {{{dp} \mathord{\left/ {\vphantom {{dp} {dy}}} \right. \kern-\nulldelimiterspace} {dy}} = p} \right)$ is shown in Figs. 19, 20, 21, 22 and 23. With increasing *m*, the $v(x,\,\alpha )$ and $u(x,\,\alpha )$ diminish. At $\alpha = 1,$ the $v(x,\,\alpha )$ and $u(x,\,\alpha )$ are the same in Figs. 18 and 23. It has a good impact on providing a classical or crisp solution. Figure 24 shows the $v(x,\,\alpha )$ and $u(x,\,\alpha )$ for various $\alpha$ values. As a result, when $\alpha = 1,$ the fuzzy velocity becomes a crisp velocity profile, demonstrated that the current problem is an extension of Kamran and Siddique^14^. Figures 25, 26 and 27 depict the uncertain behaviour of the TFN membership function with the triangle fuzzy plot when the values of *p*, *m*, and $\beta$ are varied. In Figs. 25 and 26, the uncertain width progressively reduces as the input parameters *m* and $\beta$ are increased, however in Fig. 27, the uncertain width suddenly grows when the value of *p* is increased. It was also discovered that when $\alpha$ grows, the $v(x,\,\alpha )$ increases and the higher drops, implying that the solutions are strong. The breadth between the $v(x,\,\alpha )$ and $u(x,\,\alpha )$ narrows as $\alpha$ grows, and at $\alpha = 1,$ they coherent with the traditional solution. The evaluation of $v(x,\,\alpha )$, mid, and $u(x,\,\alpha )$ at various x values using *p* = -0.2, $\beta = 0.3,$ and m = 0.2 are shown in Table 5. Furthermore, every fixed $\alpha {\text{-cut,}}$ fuzzy velocity profiles always shift within a particular range, and the range steadily declines as $\alpha {\text{-cut}}$ values increase.

**Table 5:** Fuzzy solution of $$v(x,\,\alpha )$$, mid and $$u(x,\,\alpha )$$ at fixed values of $$\alpha {\text{-cut}} = 0,$$$$m = 0.2,$$ $$\beta = 0.3,\,\,$$ and $$p = 0.2,$$ with distinct values of *x*.

*x*	$v\left( {x;\,\alpha } \right)$	Mid values	$u\left( {x;\,\alpha } \right)$	*x*	$v\left( {x;\,\alpha } \right)$	Mid values	$u\left( {x;\,\alpha } \right)$
− 1	− 0.007543	0	0.008772	0.1	0.086062	0.094703	0.107315
− 0.9	0.010613	0.022587	0.027628	0.2	0.084641	0.090161	0.102122
− 0.8	0.026813	0.038751	0.044535	0.3	0.081258	0.084137	0.094129
− 0.7	0.041062	0.052969	0.062262	0.4	0.075915	0.073317	0.083351
− 0.6	0.053360	0.067464	0.079412	0.5	0.068620	0.060188	0.069810
− 0.5	0.063706	0.079154	0.091083	0.6	0.059381	0.047145	0.062012
− 0.4	0.072098	0.088036	0.099953	0.7	0.048211	0.028995	0.047504
− 0.3	0.078533	0.094103	0.106015	0.8	0.035123	0.015397	0.034550
− 0.2	0.083007	0.097005	0.109264	0.9	0.020136	0.008153	0.027628
− 0.1	0.085517	0.097775	0.109697	1	− 0.000331	0.000008	0.016691

### Generalized Couette flow

The impact of $\beta$, *m*, and $\alpha$ on the $v(x,\,\alpha )$ and $u(x,\,\alpha )$ with a pressure gradient is shown in Figs. 28, 29, 30, 31, 32, 33, 34, 35, 36, 37, 38 and 39. The $v(x,\,\alpha )$ and $u(x,\,\alpha )$ drop gradually with growing $\beta$ in Figs. 28, 29, 30 and 31. The $v(x,\,\alpha )$ and $u(x,\,\alpha )$ grow steadily with upsurging *m* in Figs. 32, 33, 34 and 35. The $v(x,\,\alpha )$ and $u(x,\,\alpha )$ grow fast with swelling *p* in Figs. 36, 37, 38 and 39. Figures 32, 35, and 39 show that when $\alpha = 1,$ the $v(x,\,\alpha )$ and $u(x,\,\alpha )$ are the same. Figure 40 shows the $v(x,\,\alpha )$ and $u(x,\,\alpha )$ for various $\alpha$ values. Because the crisp velocity profile lies between the $v(x,\,\alpha )$ and $u(x,\,\alpha )$, when $\alpha = 1,$ the fuzzy velocity profile becomes crisp or classical, signifying that the current article is a modification of Kamran and Siddique^14^. Figures 41, 42 and 43 depict the uncertain behaviour of the TFN membership expressed as a function of the triangle fuzzy plot for various values of $\beta ,$ *p*, and *m*. Now Figs. 41 and 42, the ambiguous width steadily grows as the input parameters *m* and $\beta$ are raised, however in Fig. 43, the fuzzy width quickly rises as *p* is enhanced. Table 6 presents the assessment of $v(x,\,\alpha )$, mid, and $u(x,\,\alpha )$ velocity profiles at various *x* values using *p* = 0.015, *m* = 0.1 and $\beta = 0.2,$ as fixed values. Moreover, each fixed $\alpha {\text{-cut}},$ fuzzy velocity profile always shift within a particular range, and the range steadily reduces as $\alpha$ values increase.

**Table 6:** Fuzzy solution of $v\left( {x;\alpha } \right)$, mid and $u\left( {x;\alpha } \right)$ at $\alpha {\text{-cut}} = 0,$ *p* = 0.015, $\beta = 0.2,\,$ and $\,m = 0.01,$ with varous values of *x*.

x	$v\left( {x;\,\alpha } \right)$	Mid values	$u\left( {x;\,\alpha } \right)$
0	− 0.01015000	0	0.010149999
0.1	− 0.02030000	− 0.000000001	0.020300000
0.2	− 0.01545276	0.0149966013	0.04544596
0.3	0.00438940	0.044983248	0.08557708
0.4	0.03921724	0.089946595	0.14067595
0.5	0.08901401	0.149866060	0.210718105
0.6	0.15375484	0.22471308	0.295671319
0.7	0.23340559	0.31445006	0.395494524
0.8	0.327921567	0.41902902	0.51013648
0.9	0.43724582	0.53838998	0.63953414
1	0.56130727	0.67245899	0.78361071

The solutions are well-suited in the aforementioned discussions; the crisp solution is sandwiched between the fuzzy solutions (lower and upper-velocity profiles), and $\alpha$ approaching one position the fuzzy solutions are close to the crisp solution. The fuzzy velocity profile of the fluid is a better choice than the crisp or classical velocity profile of the fluid, according to the conclusion of the entire discussion. The single flow situation of fluid is represented by a crisp or classical velocity profile, but the interval flow situation is represented by a fuzzy velocity profile, which has lower and higher boundaries. In addition, the model described a new feature at various $\alpha$ values and gave accurate solution intervals (lower and upper-velocity profiles) for better dynamic analysis judgment.

**Plane Couette flow**

Figures 3, 4, 5, 6, 7, 8, 9, 10, 11, 12, 13 and Table 4.

**Plane Poiseuille flow**

Figures 14, 15, 16, 17, 18, 19, 20, 21, 22, 23, 24, 25, 26, 27 and Table 5.

**Generalized Couette flow**

Figure 28, 29, 30, 31, 32, 33, 34, 35, 36, 37, 38, 39, 40, 41, 42, 43 and Table 6.

## Conclusions

The three fundamental flow phenomena that inevitably arise in the study of fluid dynamics, especially plane Poiseuille, plane Couette, and generalised Couette flow of a non-Newtonian fluid under the impact of MHD force in a fuzzy environment, have been investigated in this work. The dimensionless governing DEs are discretized into FDEs with fuzzified BCs, and ADM is used to resolve them. When compared to previous results, the current crisp results acquired by ADM are shown to be in excellent agreement. The TFNs are utilised for uncertainties on the dynamic behaviour of the said problem. The velocity profiles (lower and higher) grow when the $\beta$ and $\alpha {\text{-cut}}$ increase, whereas the fuzzy velocity profile decrease as the *m* increases in three flow situations. The range of predicted lower and upper-velocity profiles is dependent on the $\alpha {\text{-cut,}}$ according to the findings. The end outcome is always an envelope of solutions with a crisp solution in the middle. As a result, As a result, fuzzy velocity fields are the modification of the crisp velocity field of a third grade fluid flowing between two parallel plates.

## Data Availability

The datasets used and/or analysed during the current study available from the corresponding author on reasonable request.

## Appendix

$$ \begin{aligned} A_{1} & = \frac{11\alpha }{{200}},\,\,A_{2} = \frac{17\alpha - 6}{{200}},\,\,A_{3} = \frac{107\alpha - 30}{{200}},\,\,A_{4} = \frac{\alpha - 1}{{100}}, \\ {\text{B}}_{1} & = \frac{41 - 21\alpha }{{400}},\,\,{\text{B}}_{2} = \frac{53 - 33\alpha }{{400}},\,{\text{B}}_{4} = \frac{1 - \alpha }{{100}},\,{\text{B}}_{3} = \frac{347 - 207\alpha }{{400}}, \\ E & = \frac{{\left( {p + 1} \right)\left( {\alpha - 1} \right)}}{100},D = \frac{{\left( {p + 1} \right)\left( {\alpha - 1} \right) - 50p}}{100}, \\ K & = \frac{{\beta D^{5} }}{5p} + \frac{{\beta D^{3} \left( {D^{4} - \left( {p + D} \right)^{4} } \right)}}{{4p^{2} }} + \frac{{\beta D^{6} }}{5p} + \frac{{m^{2} D^{3} \left( {p + 4D + 12E} \right)}}{72p}, \\ R & = \frac{{\beta \left( {p + D} \right)^{6} }}{{60p^{3} }} + \frac{{\beta \left( {D^{4} - \left( {p + D} \right)^{4} } \right)}}{12p} - \frac{{D^{4} }}{4p} - \frac{{\beta D^{6} }}{{60p^{3} }} - m^{2} \left( {\frac{6D + p + 30E - 5p - 20D - 60E}{{720}}} \right), \\ L & = \frac{{\beta \left( {p + D} \right)^{5} }}{5p} + \frac{{\beta \left( {p + D} \right)^{6} }}{5p} + \frac{{3\beta \left( {D + p} \right)^{3} \left( {D^{4} - \left( {D + p} \right)^{4} } \right)}}{{4p^{2} }} - \frac{{\beta D^{5} }}{5p} - \frac{{\beta D^{3} \left( {D^{4} - \left( {p + D} \right)^{4} } \right)}}{{4p^{2} }} \\ & \quad - \frac{{\beta D^{6} }}{5p} - m^{2} \left[ \begin{gathered} \frac{{p^{2} \left( {2p + 3} \right)}}{180} + \frac{{7D^{2} p}}{24} + \frac{{Dp^{2} }}{6} - \frac{{4EDp + D^{3} }}{6} + \frac{{\left( {p + 4D + 12E} \right)\left( {p + D} \right)^{3} }}{72p} \hfill \\ - \frac{{D^{3} \left( {p + 4D + 12E} \right)}}{72p} + \frac{{Ep^{2} }}{4} - ED^{2} \hfill \\ \end{gathered} \right], \\ E_{1} & = \frac{{\left( {1 + p} \right)\left( {1 - \alpha } \right)}}{100},\,D_{1} = \frac{{\left( {1 + p} \right)\left( {1 - \alpha } \right) - 50p}}{100}, \\ K_{1} & = \frac{{\beta D_{1}^{5} }}{5p} + \frac{{\beta D_{1}^{3} \left( {D_{1}^{4} - \left( {p + D_{1} } \right)^{4} } \right)}}{{4p^{2} }} + \frac{{\beta D_{1}^{6} }}{5p} + \frac{{m^{2} D_{1}^{3} \left( {p + 4D_{1} + 12E_{1} } \right)}}{72p}. \\ \end{aligned} $$

$$ \begin{aligned} R_{1} & = \frac{{\beta \left( {p + D_{1} } \right)^{6} }}{{60p^{3} }} + \frac{{\beta \left( {D_{1}^{4} - \left( {p + D_{1} } \right)^{4} } \right)}}{12p} - \frac{{D_{1}^{4} }}{4p} - \frac{{\beta D_{1}^{6} }}{{60p^{3} }} - m^{2} \left( {\frac{{6D_{1} + p + 30E_{1} - 5p - 20D_{1} - 60E_{1} }}{720}} \right), \\ L_{1} & = \frac{{3\beta \left( {p + D_{1} } \right)^{3} \left( {D_{1}^{4} - \left( {p + D_{1} } \right)^{4} } \right)}}{{4p^{2} }} + \frac{{\beta \left( {p + D_{1} } \right)^{6} }}{5p} - \frac{{\beta D_{1}^{5} }}{5p} + \frac{{\beta \left( {p + D_{1} } \right)^{5} }}{5p} - \frac{{\beta D_{1}^{3} \left( {D_{1}^{4} - \left( {p + D_{1} } \right)^{4} } \right)}}{{4p^{2} }} \\ & \quad - \frac{{\beta D_{1}^{6} }}{5p} + m^{2} \left[ \begin{gathered} - \frac{{D_{1} p^{2} }}{6} - \frac{{p^{2} \left( {2p + 3} \right)}}{180} + \frac{{4E_{1} D_{1} p + D_{1}^{3} }}{6} - \frac{{7D_{1}^{2} p}}{24} + E_{1} D_{1}^{2} \hfill \\ - \frac{{\left( {p + 4D_{1} + 12E_{1} } \right)\left( {p + D_{1} } \right)^{3} }}{72p} - \frac{{E_{1} p^{2} }}{4} - \frac{{D_{1}^{3} \left( {p + 4D_{1} + 12E_{1} } \right)}}{72p} \hfill \\ \end{gathered} \right], \\ G & = \frac{{\left( {D_{2} - p} \right)^{4} - \left( {D_{2} + p} \right)^{4} }}{24p},E_{2} = \frac{{3\left( {1 + p} \right)\left( { - 1 + \alpha } \right) - 100p}}{200},\,D_{2} = \frac{{\left( {1 + p} \right)\left( {1 - \alpha } \right)}}{200}, \\ V & = \frac{{ - \beta \left( {p + D_{2} } \right)^{6} + \beta \left( {p - D_{2} } \right)^{6} }}{{30p^{3} }} - m^{2} \left( {\frac{p}{180} + \frac{{E_{2} }}{6} - \frac{{p + 12E_{2} }}{12}} \right) + 12\beta F, \\ F & = \frac{{\left( {D_{2} + p} \right)^{4} + \left( {D_{2} - p} \right)^{4} }}{24p}, \\ Z & = \beta G + \frac{{7m^{2} D_{2} }}{90} + \frac{{\beta \left( {p + D_{2} } \right)^{6} - \beta \left( {p - D_{2} } \right)^{6} }}{{30p^{2} }}, \\ W & = \frac{{2G\left( {D_{2} + p} \right)^{3} + 2G\left( { - D_{2} + p} \right)^{3} }}{3p} + \frac{{ - 2\beta \left( {D_{2} + p} \right)^{6} - 2\beta \left( { - D_{2} + p} \right)^{6} }}{3p} - m^{2} \left[ \begin{gathered} \frac{{p^{3} }}{9} + \frac{{7pD_{2}^{2} }}{6} + 2E_{2} D_{2}^{2} \hfill \\ - \frac{{D_{2} \left( {p + D_{2} } \right)^{3} + D_{2} \left( {p - D_{2} } \right)^{3} }}{9p} \hfill \\ \end{gathered} \right], \\ W_{1} & = \frac{{2G\left( { - D_{2} + p} \right)^{3} - 2G\left( {p + D_{2} } \right)^{3} }}{3p} + \frac{{2\beta \left( {D_{2} + p} \right)^{6} - 2\beta \left( { - D_{2} + p} \right)^{6} }}{3p} - m^{2} \left[ \begin{gathered} \frac{{2p^{2} D_{2} }}{3} + \frac{{4p^{2} E_{2} }}{3} + 2E_{2} D_{2}^{2} + 2pE_{2} D_{2} \hfill \\ + \frac{{D_{2} \left( {p + D_{2} } \right)^{3} - D_{2} \left( {p - D_{2} } \right)^{3} }}{9p} + \frac{{2D_{2}^{3} }}{3} \hfill \\ \end{gathered} \right], \\ G_{1} & = \frac{{\left( { - p + D_{3} } \right)^{4} - \left( {p + D_{3} } \right)^{4} }}{24p}, \\ D_{3} & = \frac{{\left( {\alpha - 1} \right)\left( {1 + p} \right)}}{200},\,\,E_{3} = \frac{{3\left( {p + 1} \right)\left( { - \alpha + 1} \right) - 100p}}{200}, \\ V_{1} & = \frac{{ - \beta \left( {p + D_{3} } \right)^{6} + \beta \left( {p - D_{3} } \right)^{6} }}{{30p^{3} }} + 12\beta F_{1} - m^{2} \left( {\frac{p}{180} + \frac{{E_{3} }}{6} - \frac{{p + 12E_{3} }}{12}} \right), \\ Z_{1} & = \frac{{\beta \left( {p + D_{3} } \right)^{6} - \beta \left( {p - D_{3} } \right)^{6} }}{{30p^{2} }} + \beta G_{1} + \frac{{7m^{2} D_{3} }}{90}, \\ F_{1} & = \frac{{\left( {D_{3} + p} \right)^{4} + \left( {D_{3} - p} \right)^{4} }}{24p}, \\ W_{3} & = \frac{{2G_{1} \left( {D_{3} + p} \right)^{3} + 2G_{1} \left( { - D_{3} + p} \right)^{3} }}{3p} + \frac{{ - 2\beta \left( {p + D_{3} } \right)^{6} - 2\beta \left( { - D_{3} + p} \right)^{6} }}{3p} - m^{2} \left[ \begin{gathered} \frac{{p^{3} }}{9} + \frac{{7pD_{3}^{2} }}{6} + 2E_{3} D_{3}^{2} \hfill \\ - \frac{{D_{3} \left( {p + D_{3} } \right)^{3} + D_{3} \left( {p - D_{3} } \right)^{3} }}{9p} \hfill \\ \end{gathered} \right], \\ W_{2} & = \frac{{2\beta \left( {D_{3} + p} \right)^{6} - 2\beta \left( { - D_{3} + p} \right)^{6} }}{3p} + \frac{{2G_{1} \left( { - D_{3} + p} \right)^{3} - 2G_{1} \left( {p + D_{3} } \right)^{3} }}{3p} - m^{2} \left[ \begin{gathered} \frac{{4p^{2} E_{3} }}{3} + 2pE_{3} D_{3} + \frac{{2p^{2} D_{3} }}{3} + 2E_{3} D_{3}^{2} \hfill \\ + \frac{{2D_{3}^{3} }}{3} + \frac{{D_{3} \left( {D_{3} + p} \right)^{3} - D_{3} \left( { - D_{3} + p} \right)^{3} }}{9p} \hfill \\ \end{gathered} \right]. \\ \end{aligned} $$

## References

[1] Truesdell C, Noll W (2004). The Non-linear Field's Theories of Mechanics.

[2] Rajagopal KR (1980). On the stability of third grade fluids. Arch Mech..

[3] Rajagopal KR (1980). Thermodynamics and stability of fluids of third grade. Proc R Soc Lond A.

[4] Siddiqui AM, Mahmood R, Ghori QK (2008). Homotopy perturbation method for thin film flow of a third-grade fluid down an inclined plane. Chaos Solitons Fractals.

[5] Hayat T, Ellahi R, Mahomed FM (2008). Exact solutions for thin film flow of a third-grade fluid down an inclined plane. Chaos Solitons Fractals.

[6] Sajid M, Hayat T (2008). The application of Homotopy analysis method to thin film flows of a third order fluid. Chaos Solitons Fractals.

[7] Shah RA, Islam S, Zeb M, Ali I (2011). Optimal homotopy asymptotic method for thin film flows of a third order fluid. J. Adv. Res. Sci. Comput..

[8] Siddiqui AM, Farooq AA, Haroon T, Rana MA, Babcock BS (2012). Application of He's Variational Iterarion method for solving thin film flow problem arising in Non-Newtonian fluid mechanics. World J. Mech..

[9] Yürüsoy M, Pakdemirli M, Yilbas BS (2008). Perturbation solution for a third-grade fluid flowing between parallel plates. Proc. Inst. Mech. Eng. C J. Mech. Eng. Sci..

[10] Natarov SI, Conrad CP (2012). The role of Poiseuille flow in creating depth-variation of asthenospheric shear. Geophys. J. Int..

[11] Hayat T, Khan M, Ayub M (2004). Couette and Poiseuille flows of an Oldroyd 6-constant fluid with magnetic field. J. Math. Anal. Appl..

[12] Hayat T, Naz R, Sajid M (2010). On the homotopy solution for Poiseuille flow of a fourth grade fluid. Commun. Nonlinear Sci. Numer. Simul..

[13] Chinyoka T, Makinde OD (2011). Analysis of transient generalized Couette flow of a reactive variable viscosity third-grade liquid with asymmetric convective cooling. Math. Comput. Model.

[14] Kamran M, Siddique I (2017). MHD Couette and Poiseuille flow of a third grade fluid. Open J. Math. Anal..

[15] Khan M, Fetecau C, Hayat T (2007). MHD transient flows in a channel of rectangular cross-section with porous medium. Phys. Lett. A.

[16] Hayat T, Haroon T, Asghar S, Siddiqui AM (2003). MHD flow of a third-grade fluid due to eccentric rotations of a porous disk and a fluid at infinity. Int. J. Non Linear Mech..

[17] Hayat T, Hutter K, Asghar S, Siddiqui AM (2002). MHD flows of an Oldroyd-B fluid. Math. Comput. Model..

[18] Islam, S. Homotopy perturbations analysis of couette and poiseuille flows of a third-grade fluid with magnetic field. *Sci. Int.* **22**(3) (2010).

[19] Adomian G (1992). A review of the decomposition method and some recent results for nonlinear equations. Math. Comput. Model..

[20] Adomian G (1994). Solving Frontier Problems of Physics: The Decomposition Method.

[21] Cherruault Y, Adomian G (1993). Decomposition method: A new proof of convergence. Math. Comput. Model..

[22] Siddiqui AM, Hameed M, Siddiqui BM, Ghori QK (2010). Use of Adomian decomposition method in the study of parallel plate flow of a third grade fluid. Commun. Nonlinear Sci. Numer. Simul..

[23] Pirzada UM, Vakaskar DC (2015). Solution of fuzzy heat equations using adomian decomposition method. Int. J. Adv. Appl. Math. Mech..

[24] Paripour M, Hajilou E, Heidari H (2014). Application of Adomian decomposition method to solve hybrid fuzzy differential equations. J. Taibah Univ. Sci..

[25] Siddiqui AM, Haroon T, Bhatti S, Ansari AR (2010). A comparison of the adomian and homotopy perturbation methods in solving the problem of squeezing flow between two circular plates. Math. Model. Anal..

[26] Biswal U, Chakraverty S, Ojha BK (2020). Natural convection of nanofluid flow between two vertical flat plates with imprecise parameter. Coupled Syst. Mech..

[27] Chang SS, Zadeh LA, Zadeh LA (1996). On fuzzy mapping and control. Fuzzy Sets, Fuzzy Logic, and Fuzzy Systems: Selected Papers.

[28] Dubois D, Prade H (1978). Operations on fuzzy numbers. Int. J. Syst. Sci..

[29] Seikala S (1987). On the fuzzy initial value problem. Fuzzy Sets Syst..

[30] Kaleva O (1987). Fuzzy differential equations. Fuzzy Sets Syst..

[31] Gasilov N, Amrahov SE, Fatullayev AG (2011). A geometric approach to solve fuzzy linear systems of differential equations. Appl. Math. Inf. Sci..

[32] Khastan A, Nieto JJ (2010). A boundary value problem for second order fuzzy differential equations. Nonlinear Anal..

[33] Nadeem M, Siddique I, Jarad F, Jamil RN (2021). Numerical study of MHD third-grade fluid flow through an inclined channel with ohmic heating under fuzzy environment. Math. Probl. Eng..

[34] Biswal U, Chakraverty S, Ojha BK, Hussein AK (2021). Study of Jeffery-Hamel flow problem for nanofluid with fuzzy volume fraction using double parametric based Adomian decomposition method. Int. Commun. Heat Mass Transf..

[35] El Allaoui, A., Melliani, S., & Chadli, L. S. A mathematical fuzzy model to giving up smoking. In *IEEE 6th International Conference on Optimization and Appication (ICOA)* 1–6 (2020).

[36] Zulqarnain RM, Xin XL, Siddique I, Asghar Khan W, Yousif MA (2021). TOPSIS method based on correlation coefficient under pythagorean fuzzy soft environment and its application towards green supply chain management. Sustainability.

[37] Zulqarnain RM, Siddique I, Ali R, Jarad F, Samad A, Abdeljawad T (2021). Neutrosophic hypersoft matrices with application to solve multiattributive decision-making problems. Complexity.

[38] Zulqarnain RM, Siddique I, Ali R, Pamucar D, Marinkovic D, Bozanic D (2021). Robust aggregation operators for intuitionistic fuzzy hypersoft set with their application to solve MCDM problem. Entropy.

[39] Nadeem M, Elmoasry A, Siddique I, Jarad F, Zulqarnain RM, Alebraheem J, Elazab NS (2021). Study of triangular fuzzy hybrid nanofluids on the natural convection flow and heat transfer between two vertical plates. Comput. Intell. Neurosci..

[40] Siddique I, Zulqarnain RM, Nadeem M, Jarad F (2021). Numerical simulation of MHD couette flow of a fuzzy nanofluid through an inclined channel with thermal radiation effect. Comput. Intell. Neurosci..

[41] Nadeem, M., Siddique, I., Ali, R., Alshammari, N., Jamil, R. N., Hamadneh, N., & Andualem, M. Study of third-grade fluid under the fuzzy environment with Couette and Poiseuille flows. *Math. Probl. Eng.* (2022).

[42] Nadeem M, Siddique I, Awrejcewicz J, Bilal M (2022). Numerical analysis of a second-grade fuzzy hybrid nanofluid flow and heat transfer over a permeable stretching/shrinking sheet. Sci. Rep..

